# Glitazone Treatment and Incidence of Parkinson’s Disease among People with Diabetes: A Retrospective Cohort Study

**DOI:** 10.1371/journal.pmed.1001854

**Published:** 2015-07-21

**Authors:** Ruth Brauer, Krishnan Bhaskaran, Nishi Chaturvedi, David T. Dexter, Liam Smeeth, Ian Douglas

**Affiliations:** 1 Non-communicable Disease Epidemiology, London School of Hygiene & Tropical Medicine, London, United Kingdom; 2 Institute of Cardiovascular Sciences, University College London, London, United Kingdom; 3 Centre for Neuroinflammation & Neurodegeneration, Division of Brain Sciences, Faculty of Medicine, Imperial College, London, United Kingdom; Mount Sinai School of Medicine, UNITED STATES

## Abstract

**Background:**

Recent in vitro and animal experiments suggest that peroxisome proliferation-activated receptor gamma (PPARɣ) agonist medications, such as antidiabetic glitazone (GTZ) drugs, are neuroprotective in models of Parkinson’s disease (PD). These findings have not been tested in humans. We hypothesized that individuals prescribed GTZ drugs would have a lower incidence of PD compared to individuals prescribed other treatments for diabetes.

**Methods and Findings:**

Using primary care data from the United Kingdom Clinical Practice Research Datalink (CPRD), we conducted a retrospective cohort study in which individuals with diabetes who were newly prescribed GTZ (GTZ-exposed group) were matched by age, sex, practice, and diabetes treatment stage with up to five individuals prescribed other diabetes treatments (other antidiabetic drug-exposed group). Patients were followed up from 1999 until the first recording of a PD diagnosis, end of observation in the database, or end of the study (1 August 2013). An incidence rate ratio (IRR) was calculated using conditional Poisson regression, adjusted for possible confounders. 44,597 GTZ exposed individuals were matched to 120,373 other antidiabetic users. 175 GTZ-exposed individuals were diagnosed with PD compared to 517 individuals in the other antidiabetic drug-exposed group. The incidence rate (IR) of PD in the GTZ-exposed group was 6.4 per 10,000 patient years compared with 8.8 per 10,000 patient years in those prescribed other antidiabetic treatments (IRR 0.72, 95% confidence interval [CI] 0.60–0.87). Adjustments for potential confounding variables, including smoking, other medications, head injury, and disease severity, had no material impact (fully adjusted IRR 0.75, 0.59–0.94). The risk was reduced in those with current GTZ prescriptions (current GTZ-exposed IRR 0.59, 0.46–0.77) but not reduced among those with past prescriptions (past GTZ-exposed IRR 0.85, 0.65–1.10). Our study only included patients with diabetes who did not have a PD diagnosis when they were first prescribed GTZ, and thus, it cannot establish whether GTZ use prevents or slows the progression of PD.

**Conclusions:**

In patients with diabetes, a current prescription for GTZ is associated with a reduction in incidence of PD. This suggests PPAR gamma pathways may be a fruitful drug target in PD.

## Abstract 2

In a retrospective cohort study, Ruth Brauer and colleagues examine the association between treatment with glitazone and incidence of Parkinson's disease.

## Editors' Summary

**Background:**

Parkinson’s disease (PD) is a common, progressive neurological disease. The condition is caused by the gradual loss of nerve cells that normally produce dopamine, a neurotransmitter that regulates the body’s movements. PD does not usually begin to develop until people are around 60 years old, although it can sometimes affect younger people. Its symptoms, which develop slowly, include tremor (trembling of the hands, legs, arm, jaw, and face), slow movement, and rigidity (muscle stiffness). As these symptoms worsen, affected individuals may have trouble walking, speaking, swallowing, and sleeping, and they may become depressed. No one has found a way to halt the loss of dopamine-producing nerve cells yet, but medications that replace or mimic the lost dopamine can reduce the severity of these symptoms. PD does not directly kill people, but it puts a great strain on the body that can make affected individuals vulnerable to life-threatening infections. Nevertheless, these days, many people with PD have a normal or near-normal life expectancy.

**Why Was This Study Done?:**

Recent experiments suggest that drugs that bind to the peroxisome proliferation-activated receptor gamma (PPARγ agonist medications) may be neuroprotective (prevent nerve cell loss) in animal models of PD. PPARγ regulates how the body uses fats and sugars, and PPARγ agonist medications such as rosiglitazone and pioglitazone (glitazone [GTZ] drugs) are used to treat people with diabetes, a condition characterized by high levels of sugar in the blood. It is not known, however, whether GTZ drugs provide protection against PD in people. In this retrospective cohort study, the researchers investigate whether individuals with diabetes exposed to GTZ drugs have a lower incidence of PD than individuals using other antidiabetic drugs. A retrospective cohort study uses data already collected on a group (cohort) of people to look for associations between specific characteristics such as use of a drug and outcomes.

**What Did the Researchers Do and Find?:**

The researchers identified 44,597 patients with diabetes who were newly exposed to GTZ drugs in the UK Clinical Practice Research Datalink, a database that contains primary care data on more than 13 million individuals. They matched each patient with up to five users of other antidiabetic drugs (120,373 controls) who were similar in terms of age, sex, attendance at the same primary care practice, and diabetes treatment stage; people with diabetes usually initially take a single drug but often switch to a different drug or to a combination of drugs as their disease progresses. Finally, the researchers followed up the entire cohort from January 1999 (when glitazones were introduced to treat diabetes) until diagnosis of PD, the end of inclusion in the database, or the end of the study (August 2013). During follow up, 175 glitazone users and 517 non-glitazone users were diagnosed with PD. The incidence rates of PD among glitazone-using individuals and among users of other antidiabetic treatments were 6.4 per 10,000 patient years and 8.8 per 10,000 patient years, respectively (an incidence rate ratio [IRR] of 0.72; an IRR compares the rate of occurrence of new cases of a disease in two groups of people). Adjustment for potential confounding variables (characteristics that might affect an individual’s likelihood of developing PD) such as smoking and head injury did not alter the IRR. Notably, the risk of PD was reduced among current users of GTZ drugs but not among past users.

**What Do These Findings Mean?:**

These findings indicate that, among people with diabetes, current (but not past) GTZ use is associated with a 28% lower rate of clinical presentation of PD compared to the use of other antidiabetic drugs. Because the study only included patients with diabetes who did not have a PD diagnosis when they started GTZ treatment, these findings cannot establish whether GTZ use is associated with prevention of PD or with slower progression of the disease. Moreover, the accuracy of these findings may be affected by misdiagnosis of PD and misclassification of exposure periods to various antidiabetic drugs in the database and by residual confounding. Finally, these findings may not be applicable to people without diabetes. Importantly, the researchers do not recommend that GTZ drugs (which have been associated with some serious side effects) be used as a treatment for PD. Rather, they suggest that the pathways in which PPARγ is involved might contain potential drug targets for PD and should be investigated in future research.

**Additional Information:**

This list of resources contains links that can be accessed when viewing the PDF on a device or via the online version of the article at http://dx.doi.org/10.1371/journal.pmed.1001854.

- The United States National Institute of Neurological Disorders and Stroke provides detailed information about PD (in English and Spanish), including links to US organizations that help people with PD
- The UK National Health Service Choices website provides information on all aspects of PD (including personal stories)
- The UK not-for-profit organization Parkinson's^UK^ and the US not-for-profit organization National Parkinson Foundation also provide detailed information about PD and personal stories
- The UK not-for-profit organization Healthtalk.Org provides stories about all aspects of living with PD
- MedlinePlus has links to further resources about PD and information about rosiglitazone and pioglitazone (in English and Spanish)

## Introduction

Parkinson’s disease (PD) is a common, progressive degenerative neurological disease, with a markedly increased prevalence with older age [1]. It is principally characterised by neurodegeneration of the nigrostriatal neurons and a consequent deficit in striatal dopamine content [2,3]. Until now, no effective treatments have been found to tackle directly the neurodegenerative aspect of PD.

Promising in vitro and in vivo studies with rodents show that peroxisome proliferation-activated receptor gamma (PPARɣ) agonist medications, such as the antidiabetes glitazone (GTZ) drugs pioglitazone and rosiglitazone, may have neuroprotective effects by inhibiting microglial activation [4–6]. There is one ongoing small clinical trial among patients with PD looking at disease progression (Clinicaltrials.gov registration: NCT01280123), but there are no published human data on whether GTZ drugs are protective against PD.

Utilizing electronic health records, we aimed to determine whether there is a protective association between exposure to GTZ antidiabetic medications and PD in a large primary care population of people with diabetes.

## Methods

Our study protocol was approved by the Independent Scientific Advisory Committee for MHRA database research (Independent Scientific Advisory Committee [ISAC] protocol number 13_016, February 2013). Studies using anonymised CPRD data for ISAC-approved observational research are covered by CPRD’s broad MREC ethics approval, without the need for specific informed consent.

### Data Source and Study Design

A cohort study was conducted among people with type 1 and 2 diabetes within the United Kingdom Clinical Practice Research Datalink (CPRD), a well-established research database of electronic health records. CPRD collects and archives the anonymised medical, laboratory, referral, and prescribing records of primary care practices. CPRD data are demographically and geographically representative of the UK population, representing around 8% of it [7,8]. This study used the medical records of patients registered at one of 680 participating practices, comprising 13,291,839 active patients in October 2013 (month of data retrieval). As primary care is free at the point of delivery, it has almost universal coverage in the UK. The database has been described in detail elsewhere [9].

### Selection of GTZ Users and the Comparison Group

The study population was drawn from the entire CPRD population, with follow-up time from the first of January 1999 onwards, when rosiglitazone was introduced. This was a dynamic cohort, with follow up ending at the earliest of the following: the date the patient left the practice, death date, or the latest date of data collection (1August 2013). Exposed patients were selected for inclusion if they had received any prescription for a GTZ (code list in S1 Text), with the first occurrence being at least 12 months after the patient’s start of follow up to ensure incident use. No age restrictions were applied. Each GTZ-exposed patient was matched by age (within 5 years), sex, practice, and treatment stage (see “Definition of Exposure and Treatment Stages”) with one to five patients starting other treatments for diabetes from 1999 onwards, again with at least 12 months prior registration in the database (other antidiabetic drug-exposed group). Individuals in the control pool who were eligible as potential matches for both first-line GTZ users and second-line GTZ users were removed from the potential control pool once selected as a match for first-line GTZ users. Follow up for all patients began at the first prescribing event that made them eligible for inclusion (index date). Patients in the GTZ-using group exposed to other antidiabetic drugs prior to the start of their GTZ follow up were eligible as potential matches pre-GTZ use. Potential participants with any diagnosis of PD prior to their index date were excluded. The time index used in this study was follow-up time in years after study entry. The mean follow-up time was 5.23 years (standard deviation 3.75), with a range of 0 to 14.57 years.

### Definition of Exposure and Treatment Stages

Exposure was defined as a recorded prescription for an antidiabetic agent listed in chapter 6.1 of the British National Formulary (BNF). To account for missing information regarding dose and duration of treatment, the median obtained from all other prescriptions of the same drug was imputed if no other information was available. In the primary analysis, exposure was characterized as “ever exposed” versus “never exposed” to GTZ drugs in a time-updated manner: GTZ-exposed individuals at the start of their follow-up time remained ever exposed regardless of any subsequent changes in therapy; nonusers starting a GTZ during follow up changed from never exposed to ever exposed from the date of the change.

**Fig 1:**
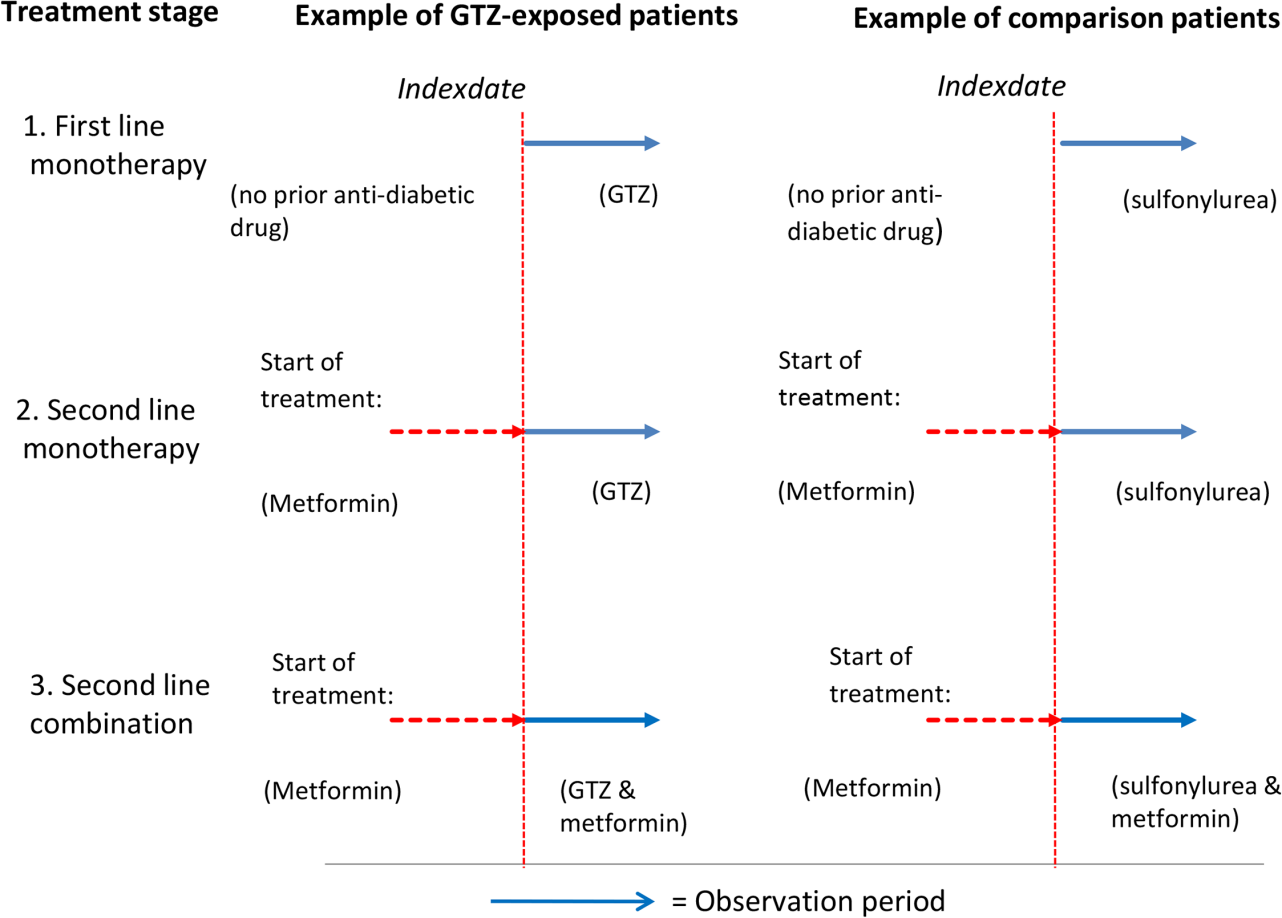
Graphical representation of the three different treatment stages: Users of GTZ drugs and other antidiabetic drugs classified according to first- or second-line mono- or combination therapy at the index date. For instance, to be a glitazone user in category (2), first-line non-GTZ treatment for diabetes was discontinued after 1999 when a GTZ was prescribed as second-line monotherapy. A patient could be matched to a GTZ user in category (2) if they had received an antidiabetic drug other than a GTZ (for example: Metformin) but stopped using their first-line antidiabetic treatment after 1999 and began taking another drug (for example: Sulfonylurea) as monotherapy after at least 12 months of registered follow-up.

GTZ-exposed and other antidiabetic drug-exposed individuals were classified according to their diabetes treatment stage at the index date as shown in Fig 1: stage (1) monotherapy with GTZ only or monotherapy with any other antidiabetic drugs, with no prior pharmaceutical treatment for diabetes received; stage (2) second-line GTZ as monotherapy or a second-line other antidiabetic drug as monotherapy, with the first-line antidiabetic drug discontinued; and stage (3) second-line GTZ in combination with one or more other antidiabetic drugs, versus any other non-GTZ antidiabetic drugs in combination of two or more drugs (see Fig 1).

### Identification of Patients with PD

The outcome for this study was the first recording of a PD diagnosis after the index date. People with a first recorded occurrence of PD were identified using clinical and referral records, using the Read code list in S2 Text. Any person with a diagnosis of PD due to known causes (such as drug-induced PD), based on the recording of codes presented in S2 Text, was censored at the date of the diagnosis.

### Statistical Methods

We measured an incidence rate ratio (IRR) for the association between GTZ exposure and incident PD using conditional Poisson regression, conditional on the matched sets to control for gender, age, practice, and treatment stage. GTZ-exposed individuals were compared with patients exposed to alternative treatments. The primary analysis followed all patients from their index date until the earliest of the following: recording of PD, transfer out/death, or last data collection date for the practice.

Adjustments for potential confounding were made in a forward stepwise manner, retaining variables in the model if they had a substantial impact on the estimated rate ratio for GTZ-exposure (a change of 10% or more) and starting with variables associated with PD incidence in univariate analyses. Additionally, we included a model adjusting for all potential confounders. Covariates that were explored at the time of entry to the study because of their potential association with both GTZ exposure and PD were current smoking, prescriptions for hormone replacement therapy and calcium channel blockers, head trauma, calendar year, alcohol consumption, and body mass index (BMI) [10–16]. A protective effect of diabetes, compared to people without diabetes, has recently been reported [17]. Our analysis was restricted to people with diabetes, but the degree of protection may be related to disease severity. As such, we included measures of severity as confounders, including HbA_1c_ level at the index date and diabetes duration to index date. In a secondary analysis, we also adjusted for time-updated age (5-year age bands, except for 50–70 years for which we used 2-year bands) and calendar year (see below).

All data management and analyses were performed using Stata 12 (StataCorp, Texas).

### Secondary Analyses

Firstly, we took into account cessation of GTZ therapy by dividing GTZ-exposed follow-up time into current and past use: users were designated as past users 180 days after their last GTZ prescription. The 180 days represent a gradual shift from full exposure to an entirely unexposed state, allowing for potential delayed use of medication. Within this post hoc time-updated analysis, a stratified analysis on length of follow up of current GTZ exposure was conducted to assess whether the treatment association varied with exposure length. Secondly, a stratified analysis was conducted to look separately at the association with pioglitazone and rosiglitazone.

### Sensitivity Analyses

We performed several sensitivity analyses:

1. Metformin has been suggested to also have a protective effect against PD [18]. To explore any potential effect modification by metformin use, we performed an analysis restricted to current or past metformin users.
2. A sensitivity analysis was conducted using a stricter definition of PD in which at least two prescriptions for an anti-PD drug were required in addition to a Read code indicating PD, to reduce the likelihood of outcome misclassification. The date of earliest recorded diagnosis remained the date of the outcome.
3. Post hoc sensitivity analyses were conducted restricted to all users of antidiabetic agents aged 40 and older; in a separate analysis, analyses were restricted to nonsmokers. Lastly, to examine the association with oral antidiabetic agents only, we excluded individuals who were prescribed insulin before or on the index date.

## Results

### Patient Characteristics

**Fig 2:**
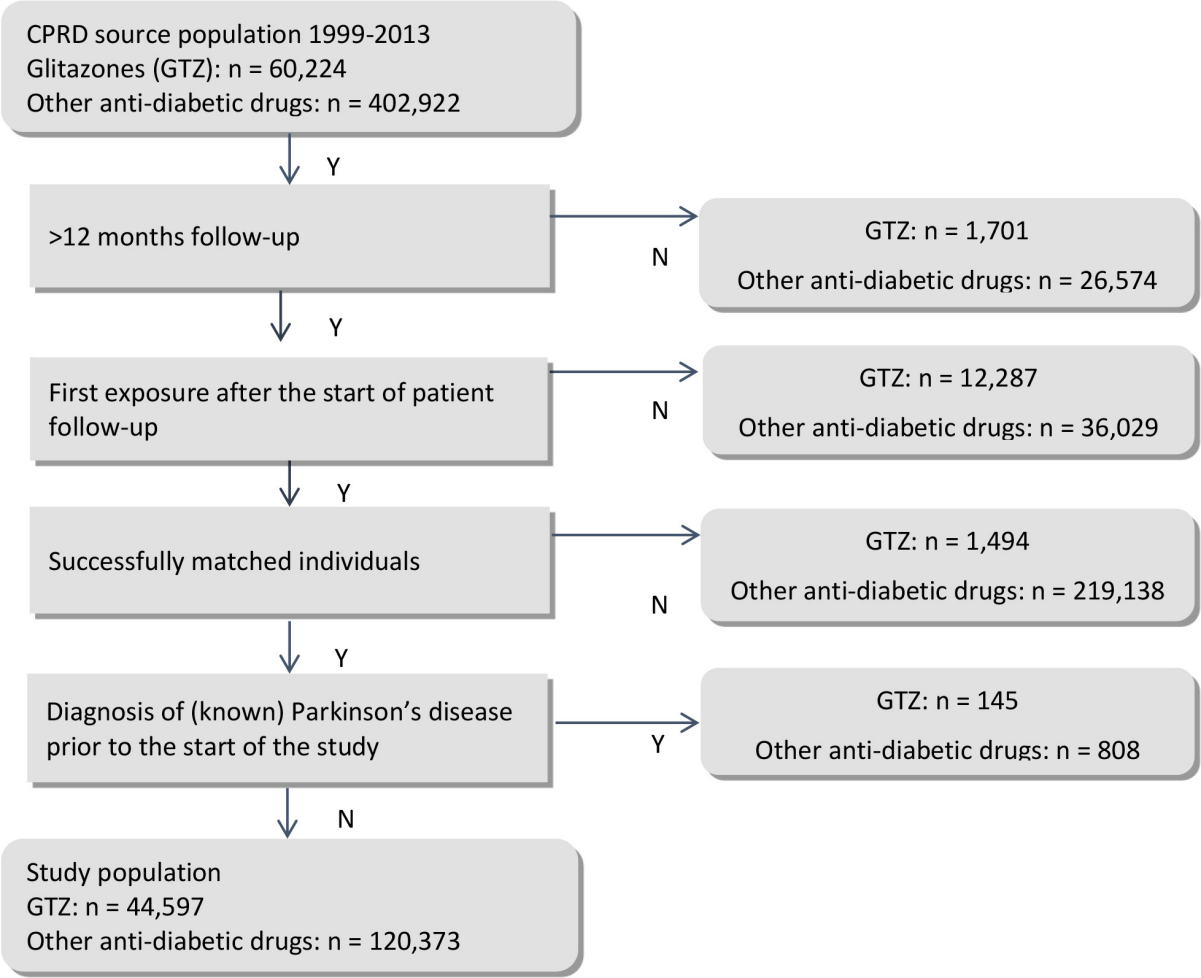
Flow chart study population: Creation of the main dataset and reasons for exclusions

Of the 60,224 patients with a recorded GTZ prescription from 1999 onwards, 44,597 met the inclusion criteria (see Fig 2). 13,988 GTZ-exposed individuals had less than 12 months of follow-up time or were exposed to GTZ drugs before the start of follow up. 1,494 GTZ-exposed individuals could not be matched to users of other antidiabetic drugs because of a lack of available matches with the same age, gender, treatment stage, and practice and were dropped from the analyses, as were 145 GTZ users diagnosed with PD before GTZ use. For the primary analysis, the GTZ-exposed individuals were matched to 120,373 other antidiabetic drug-exposed individuals. The average follow-up time of GTZ-exposed individuals and those prescribed other antidiabetic treatments from the case index date within the matched set until the overall end of follow up in CPRD was similar (6.1 versus 5.9 years). 81% of all patients in the GTZ-exposed groups (*n* = 36,341) and 79% (*n* = 95,331) of those prescribed other treatments for diabetes were followed up from the start date until the final data collection date. Four percent of all study participants (1,561 individuals exposed to GTZ drugs and 4,946 individuals exposed to other treatments for diabetes) were followed up until they transferred to another primary care practice, and the remaining patients (6,695 GTZ-exposed and 20,096 individuals exposed to other treatments for diabetes) were followed up until their recorded death date. Most of the GTZ-exposed group (94%) were prescribed second-line GTZ as dual therapy in combination with metformin (55%) or sulphonylureas (39%). The characteristics of the GTZ-exposed and the matched other antidiabetic drug-exposed group are presented in Table 1.

**Table 1:** Patient demographics and characteristics: GTZ-exposed individuals versus other antidiabetic drug-exposed individuals.

**Demographics, Characteristics**	**GTZ-exposed individuals (*n* = 44,597)**	**Other antidiabetic drug-exposed individuals (*n* = 120,373)**
**Median age (interquartile ranges)**	62.7 (53.9–71.1)	63.6 (54.4–71.9)
**Age range (years)**	7.3–98.9	8.1–100.3
**Sex, *n* (%)**		
Female	19,249 (43.2)	52,471 (43.6)
Male	25,348 (56.8)	67,902 (56.4)
**Calendar year at index date, *n* (%)**		
1999–2003	10,019 (22.5)	43,982 (36.5)
2004–2008	26,090 (58.5)	37,463 (31.1)
2009–2013	8,488 (19.0)	38,928 (32.4)
**Alcohol use at study entry, *n* (%)**		
No	6,487 (14.6)	21,302 (17.7)
Yes	30,633 (68.7)	77,281 (64.2)
Former	3,431 (7.7)	7,511 (6.2)
Excessive	2,378 (5.3)	7,135 (5.9)
Unknown	1,668 (3.7)	7,144 (5.9)
**Smoking at study entry, *n* (%)**		
Current	7,403 (16.6)	22,411 (18.6)
Former smoker	22,483 (50.4)	56,041 (46.6)
Never	14,083 (31.6)	40,085 (33.3)
Unknown	628 (1.4)	1,836 (4.5)
**BMI (kg/m** ^**2**^ **) at study entry, *n* (%)**		
<18.5	130 (0.3)	577 (0.5)
18.5–24.9	5,352 (12.0)	18,422 (15.3)
25.0–29.9	14,578 (32.7)	40,933 (34.0)
≥30	23,727 (53.2)	56,188 (46.7)
Unknown	810 (1.8)	4,253 (3.5)
**Treatment stage, *n* (%)**		
First-line monotherapy	942 (2.1)	4,317 (3.6)
Second-line monotherapy	1,768 (4.0)	3,182 (2.6)
Second-line combination	41,887 (93.9)	112,874 (93.7)
**Comorbidities, *n* (%)**		
*Head injury*	1,457 (3.3)	3,885 (3.2)
*Diabetes duration*		
0–2 years before entry study	8,979 (20.1)	32,146 (26.7)
2–5 years before entry study	12,631 (28.3)	28,584 (23.7)
5–9 years before entry study	12,308 (27.6)	28,827 (23.9)
>9 years before entry study	10,595 (23.8)	30,438 (25.3)
Diabetes diagnosis missing	84 (0.2)	378 (0.3)
*HbA*_*1c*_ *levels*		
<7.5% (<57 mmol/mol)	8,419 (18.9)	37,912 (31.5)
7.6%–8.3% (>57 mmol/mol and <66 mmol/mol)	11,810 (26.5)	26,505 (22.0)
8.4%–9.6% (>66 mmol/mol and <80 mmol/mol)	12,444 (27.9)	25,005 (20.8)
>9.6%	11,751 (26.3)	28,966 (24.1)
HbA1c missing	173 (0.4)	1,985 (1.6)
**Comedications, *n* (%)**		
*Calcium channel blockers (CCB)*		
Never	28,115 (63.0)	78,397 (65.1)
Past user: Used >6 months before index date	6,811 (15.3)	23,287 (19.4)
Current user for *<*1 year	1,182 (2.7)	3,376 (2.8)
Current user for >1 year	8,489 (19.0)	15,313 (12.7)
*Hormone replacement therapy (HRT)*		
Never	39,516 (88.6)	109,578 (91.0)
Past user: Used >6 months before index date	4,200 (9.4)	8,834 (7.3)
Current user for *<*1 year	67 (0.2)	324 (0.3)
Current user for >1 year	814 (1.8)	1,637 (1.4)

GTZ-exposed individuals were more likely than those prescribed other antidiabetic drugs to have BMI > 25 kg/m^2^ and to have higher HbA_1c_ levels at baseline. The average age of PD diagnosis in GTZ users was similar to non-GTZ users (73.2 versus 73.5 years). See S1 Table for a comparison of individuals with and without PD.

### Results Primary Analysis

The incidence rate (IR) of PD in the GTZ-using group (6.4 per 10,000 patient years, 95% confidence interval [CI] 5.5–7.4) was lower than the IR of PD in the matched comparison cohort (8.8 per 10,000 patient years, 95% CI 8.1–9.6). The IRR, accounting for age, gender, practice, and treatment stage by matching, showed strong evidence of a protective association between use of GTZ drugs and the onset of PD (IRR 0.72, 95% CI 0.60–0.87, *p*-value < 0.001). The results of the primary analysis are presented in Table 2. A similar association was found when adjusting for all potential confounders (fully adjusted IRR 0.75, 0.59–0.94, *p*-value = 0.011; see Table 3).

**Table 2:** IRR of PD: GTZ-exposed group versus the other antidiabetic drug-exposed group.

Type of Analysis	Group	Person Years	PD Cases	Adjusted IRR* (95% CI)
**Primary analysis:**	Other antidiabetic drug-exposed group	587,767	517	1
	GTZ-exposed group	273,435	175	0.72 (0.60–0.87)
**Secondary analyses:**				
Time-updated follow-up periods:				
*Current*	GTZ-exposed group	156,532	89	0.59 (0.46–0.77)
*Current* 0–2 years**	GTZ-exposed group	16,910	8	0.53 (0.25–1.12)
*Current* 2–3 years	GTZ-exposed group	13,945	9	0.56 (0.27–1.17)
*Current* >3 years	GTZ-exposed group	125,678	72	0.61 (0.46–0.81)
Past	GTZ-exposed group	116,895	86	0.85 (0.65–1.10)
Analysis stratified by type of GTZ:				
*Pioglitazone*	Other antidiabetic drug-exposed group	250,996	180	1
	GTZ-exposed group	94,218	57	0.89 (0.65–1.24)
*Rosiglitazone*	Other antidiabetic drug-exposed group	336,771	337	1
	GTZ-exposed group	179,207	118	0.65 (0.52–0.82)
**Sensitivity analyses:**				
Analysis with secondary outcome definition	Other antidiabetic drug-exposed group	587,340	398	1
	GTZ-exposed group	273,508	148	0.82 (0.66–1.00)
Analysis stratified by metformin use: *Metformin*	Other antidiabetic drug-exposed group	434,566	397	1
	GTZ-exposed group	253,325	158	0.64 (0.52–0.79)
Analysis age-restricted cohort (>40 years)	Other antidiabetic drug-exposed group	553,942	515	1
	GTZ-exposed group	262,113	175	0.73 (0.60–0.87)
Analysis cohort restricted to nonsmokers	Other antidiabetic drug-exposed group	197,324	184	1
	GTZ-exposed group	86,699	52	0.69 (0.44–1.09)
Analysis cohort restricted to users of oral antidiabetic agents	Other antidiabetic drug-exposed group	460,917	426	1
	GTZ-exposed group	261,746	173	0.78 (0.64–0.95)

* Adjusted for matched variables (age, gender, practice, and treatment stage) by conditional Poisson regression analysis.

** The rate of PD for current users of GTZ was originally divided into exposed <6 months, 6–12 months, and 1–2 years, but these cells were combined to avoid small cells that could compromise anonymity. The crude rates in these three periods were all similar at between 4.36 and 4.94, so there was no suggestion of important variation in that time period.

**Table 3:** IRR of PD adjusted for potential confounders.

Main Analysis* Adjusted for:	Adjusted IRR** (95% CI)
Year at index date	0.71 (0.58–0.88)
Smoking	0.71 (0.59–0.86)
Use of alcohol	0.73 (0.60–0.89)
BMI	0.72 (0.59–0.87)
Head injury	0.72 (0.58–0.88)
Duration of diabetes	0.72 (0.59–0.87)
HbA_1c_ levels at baseline	0.76 (0.63–0.92)
Use of CCB	0.73 (0.61–0.88)
Use of HRT	0.72 (0.60–0.87)
All variables	0.75 (0.59–0.94)

* Main analysis: IRR for the association between GTZ use and incident PD using conditional Poisson regression to control for gender, age, practice, and treatment stage

A simple test for interaction by age (above and below the median age) as a post hoc analysis showed no evidence for effect modification by age (*p*-value = 0.9729).

### Results Secondary Analyses

When the follow-up time of the GTZ–exposed group was updated to differentiate between the current and past GTZ-exposed groups, a strong protective association was found for current GTZ-exposure (IRR 0.59, 95% CI 0.46–0.77, *p*-value < 0.0001), but we observed a weaker, nonsignificant association with past use (IRR 0.85, 95% CI 0.65–1.10, *p*-value = 0.209). A stratified analysis on length of follow up of current GTZ exposure suggested no important variation in rates of PD depending on duration of use (see Table 2).

The point estimate for the estimated protective association was stronger for rosiglitazone compared to other antidiabetic drugs (IRR = 0.65, 0.52–0.82, *p*-value < 0.0001) than pioglitazone compared to other antidiabetic drugs (IRR = 0.89, 0.65–1.24, *p*-value = 0.496), but numbers of events were limited in the latter group, CIs overlapped, and a formal test suggested no evidence of an interaction (*p*-value for interaction = 0.42).

### Results Sensitivity Analyses

Restricting the cohort or changing the definition of PD did not have a material impact on the main results: the association between exposure to GTZ drugs and the lower incidence of PD remained (see Table 2).

## Discussion

In a large population-based cohort study, we have shown for the first time that in individuals with diabetes, a prescription for GTZ is associated with a 28% lower rate of clinical presentation of PD compared to those prescribed other antidiabetic agents. The estimated association was similar when established risk factors, such as age and smoking, and possible risk factors, such as head injury and BMI, were adjusted for. Results allowing for changes in GTZ exposure showed strong evidence of a protective association between current GTZ exposure and PD compared with other antidiabetic drug exposure (IRR 0.59, 95% CI 0.46–0.77). No significant association was found for past exposure (IRR 0.85, 95% 0.65–1.10). Our results suggest that the protective association is limited to periods of GTZ treatment, with little or no longer-lasting benefit. This is supported by animal study data in which withdrawal of PPARγ therapy negates any neuroprotective effect [19].

To our knowledge, this is the first population-based investigation of the relationship between treatment with GTZ drugs and the incidence of PD. A clinical trial is currently investigating pioglitazone as a therapy to slow disease progression in early-stage PD in 216 participants (Clinicaltrials.gov registration: NCT01280123), but this trial is not designed to assess an effect on disease incidence. Our study provides unique evidence to support further investigation of GTZ use in PD.

Evidence was found of an association between the use of both pioglitazone and rosiglitazone and the onset of PD. Rosiglitazone was introduced to the market in 1999 but was eventually suspended because of cardiovascular safety concerns [20]. However, in late 2013 the FDA removed the prescribing and dispensing restrictions for rosiglitazone medicines that were put into place in 2010 [21]. Whilst pioglitazone use has waned because of concerns over the risk of bladder cancer, recent studies show that the association may be partly mediated by proteinuria testing [22,23].

Stratifying by year of GTZ initiation did not change the main results, as strong associations were found for relatively short follow-up periods of use.

Regarding the mechanisms of neuroprotection, glitazones have been shown to reduce the expression of matrix metalloproteinases (MMP 3 and 9) by traumatised neurons, which are known microglial activators as well as a direct anti-inflammatory on microglia [5]. GTZ drugs have been shown to have a number of other neuroprotective properties in addition to their anti-inflammatory effects. GTZ treatment, through its ability to up-regulate PGC-1alpha expression, has been shown to increase brain mitochondrial biogenesis and the expression of antioxidant enzymes and antiapoptotic factors [5,24–27]. Underexpression of peroxisome proliferator-activated receptor-gamma coactivator (PGC)-1α has been identified as a potential link for manifestations of defects in mitochondrial biogenesis and energetics [28]. Selective loss of dopamine neurons in the substantia nigra can be reversed by PGC-1α expression in both in vitro and in vivo models [28,29]. This could be potentially important since mitochondrial deficits and oxidative stress have also been implicated in the pathogenesis of PD. Additional peripheral mechanisms of neuroprotection may be through adiponectin, or adipocyte complement-related protein 30 (acrp30), which is elevated following the administration of PPAR agonists and has anti-inflammatory properties in its own right [5]. Short-term treatment with GTZ drugs in animal models produced dramatic neuroprotection, larger than what is often observed by many neuroprotective compounds. Hence, even short-term treatment with GTZ drugs in the presymptomatic phase of PD, when the nigrostriatal system will be fairly intact, may be able to break the cycle of neurodegeneration by inhibiting the neuroinflammation process and preventing or slowing disease onset [19].

### Strengths and Limitations

Our cohort included a large study population of >160,000 diabetes patients from a health care database with representative population coverage, in a setting with near-universal access to medical care across the demographic and socioeconomic spectrum. As the electronic primary care record is used to generate patient prescriptions, medication data are complete and accurate. Repeat prescriptions are triggered by the patient; thus, our measures of drug use likely reflect actual use. Misclassification of exposure periods could have occurred if patients were not adhering completely to prescribed medicines, but diabetes is a serious condition for which patients are likely to be motivated to take their medication, and any such misclassification is likely to have been nondifferential with respect to PD status. Similarly, confounders such as smoking status and alcohol use will be subject to some imprecision, but this is unlikely to be differential according to GTZ user status.

Diagnoses of PD in CPRD have a positive predictive value of 81% (primary definition) to 90% (secondary definition) [13,30]. The results of a secondary analysis with a more stringent definition of PD (clinical diagnosis plus at least two prescriptions for an anti-PD drug) gave similar results (0.82 versus 0.72 in the primary analysis). Given the overall low prevalence of PD, it is unlikely that a substantial number of patients were missed. As we did not take into account any future treatment breaks or changes, we may have misclassified the exposure status of individuals in our primary analysis. However, in a secondary analysis we allowed for treatment changes and breaks (current versus past exposure to GTZ drugs) and found a strong association with current use. Moreover, in a post hoc analysis, we compared current GTZ exposure with current exposure to other treatments for diabetes, allowing for treatment breaks in both groups: the strong association with current GTZ exposure remained (IRR 0.56, 95% CI 0.43–0.72). Any misclassification in our primary analysis is likely to have been nondifferential and could have caused bias towards the null, or a potential underestimation of the protective association.

The onset of PD in the context of the current study refers to the first recorded clinical diagnosis of PD in the CPRD. Patients are likely to have experienced symptoms before the date of their clinical diagnosis. We compared the age of onset of PD between GTZ users and nonusers but found no difference in average age, suggesting that GTZ drug do not simply delay the appreciation of symptoms. A key clinical consideration is whether GTZ can slow PD progression after onset. Unfortunately, we cannot address this question with these data, as objective markers of PD progression do not exist and measures of symptom progression are subjective and poorly captured in the primary care record. Furthermore, increases in dosage of anti-PD medication or the addition of added anti-PD therapies, e.g., catechol-O-methyltransferase (COMT) inhibitors (used in patients with motor fluctuations), are not reliable markers of disease progression. When we attempted to compare the time to first PD treatment after the clinical PD diagnosis, we found that a large number of GTZ users stopped their GTZ before PD diagnosis. GTZ drugs, like all medications, have known side effects, and we are not making any recommendations about their use in treating PD, since the balance of any potential benefits and risks is entirely unknown at this stage.

Whilst we tried to select comparator groups that were as similar as possible to the GTZ users, we cannot rule out unknown or unmeasured differences between the groups. The main reason for nonmatches was the lack of similar patients per treatment stage, and this is a matching criterion that cannot be weakened in a meaningful way. GTZ drugs are most often prescribed as a second-line treatment to patients who have responded less successfully to other treatments for diabetes. We matched these patients to other users of second-line treatment at the date of receipt of the add-on treatment. We controlled for the date of onset of diabetes and HbA1c levels in a fully adjusted analysis. As the results were very similar to the results of the primary analysis, we do not believe that GTZ patients in this study had a different underlying risk of developing PD compared with users of other antidiabetic drugs. Adjusting for other known predictors of PD (most notably smoking) did not appreciably change the estimated association with GTZ use. As none of the potential risk factors were strongly associated with the decision to prescribe a GTZ, only the matching variables were retained in the final model.

The study was conducted entirely within a patient population with diabetes, which may affect the generalisability of our results. However, adjusting for duration of diabetes did not have an effect on the results of the main analysis (IRR 0.72, 95% CI 0.59–0.87), and we believe that it is unlikely that any pharmacological effect of GTZ drugs relevant to PD is limited to people with diabetes only.

## Conclusion

We found strong evidence for a protective association between GTZ exposure and the IR of clinical presentation with PD in humans, consistent with previous evidence from animal and in vitro studies. Our findings indicate that interventions based on the same mechanisms as PPARγ agonist activity may be fruitful targets for future research in PD.

## Supporting Information

S1 STROBE Checklist Checklist of items that should be included in reports of cohort studies. (DOC)

S1 Protocol ISAC application form. Protocol for research using the CPRD.
(DOC)

S1 Table Patient demographics and characteristics: Individuals diagnosed with PD versus individuals not diagnosed with PD. (DOC)

S1 Text Codes used to identify GTZ exposure. (DOC)

S2 Text Codes used to identify PD: PD with no identified cause and PD with identified causes. (DOC)

S3 Text Codes used for variables listed in Table 1. (DOCX)

## Abbreviations:

acrp30: adipocyte complement-related protein 30
BMI: body mass index
BNF: British National Formulary
CCB: calcium channel blockers
CI: confidence interval
COMT: catechol-O-methyltransferase
CPRD: Clinical Practice Research Datalink
GTZ: glitazone
HRT: hormone replacement therapy
IR: incidence rate
IRR: incidence rate ratio
ISAC: Independent Scientific Advisory Committee
MMP: matrix metalloproteinase
PD: Parkinson’s disease
PGC: peroxisome proliferator-activated receptor-gamma coactivator
PPARɣ: peroxisome proliferation-activated receptor gamma
