# Supplementary material for: Glitazone Treatment and Incidence of Parkinson’s Disease among People with Diabetes: A Retrospective Cohort Study
Source: PLoS Med. 2015 Jul 21;12(7):e1001854. doi: 10.1371/journal.pmed.1001854 (PMC4511413; doi:10.1371/journal.pmed.1001854)
Supplement: S1 STROBE Checklist — (DOC) [file pmed.1001854.s001.doc]

STROBE Statement—Checklist of items that should be included in reports of ***cohort studies***

|  | Item No | Recommendation |
| --- | --- | --- |
| **Title and abstract** | x | (*a*) Indicate the study’s design with a commonly used term in the title or the abstract  **Abstract: Paragraph 2 *(Methods and Findings)*** |
|  |  | (*b*) Provide in the abstract an informative and balanced summary of what was done and what was found  **Abstract: Paragraph 1-4** |
| Introduction | | |
| Background/rationale | x | Explain the scientific background and rationale for the investigation being reported  **Introduction: Paragraph 1-3** |
| Objectives | X | State specific objectives, including any prespecified hypotheses  **Introduction, Paragraph 3** |
| Methods | | |
| Study design | X | Present key elements of study design early in the paper  **Methods: *Selection of GTZ users and the comparison group*, *Definition of exposure and treatment stages.*** |
| Setting | X | Describe the setting, locations, and relevant dates, including periods of recruitment, exposure, follow-up, and data collection  **Methods: *Data source and study design, Selection of GTZ users and the comparison group*** |
| Participants | X | (*a*) Give the eligibility criteria, and the sources and methods of selection of participants. Describe methods of follow-up  **Methods: *Data source and study design, Selection of GTZ users and the comparison group, Definition of exposure and treatment stages, Identification of patients with Parkinson’s disease*  and Figure 1** |
|  |  | (*b*) For matched studies, give matching criteria and number of exposed and unexposed  **Methods: *Statistical Methods*** |
| Variables | X | Clearly define all outcomes, exposures, predictors, potential confounders, and effect modifiers. Give diagnostic criteria, if applicable  **Methods: *Definition of exposure and treatment stages, Identification of patients with Parkinson’s disease & Statistical Methods*. See also: Supporting Information S1 Text & S2 Text** |
| Data sources/ measurement | X | For each variable of interest, give sources of data and details of methods of assessment (measurement). Describe comparability of assessment methods if there is more than one group  **Methods: *Data source and study design, Definition of exposure and treatment stages, Identification of patients with Parkinson’s disease & Statistical Methods*.** |
| Bias | X | Describe any efforts to address potential sources of bias  **Methods: *Statistical Methods*, *Secondary and Sensitivity analyses*** |
| Study size | X | Explain how the study size was arrived at  **Methods: *Selection of GTZ users and the comparison group*** *(nb. no pre-defined study size, all eligible individuals included)* |
| Quantitative variables | X | Explain how quantitative variables were handled in the analyses. If applicable, describe which groupings were chosen and why  **Methods: *Definition of exposure and treatment stages, Identification of patients with Parkinson’s disease; and Statistical Methods*** |
| Statistical methods | X | (*a*) Describe all statistical methods, including those used to control for confounding  **Methods: *Statistical methods*** |
|  |  | (*b*) Describe any methods used to examine subgroups and interactions  **Methods: *Statistical methods, Secondary and Sensitivity analyses*** |
|  |  | (*c*) Explain how missing data were addressed  **Methods: *Definition of exposure and treatment stages,*  *Sensitivity analyses*** |
|  |  | (*d*) If applicable, explain how loss to follow-up was addressed  **Results: *Patient characteristics (Paragraph 1)*** |
|  |  | (*e*) Describe any sensitivity analyses  **Methods: *Sensitivity analyses*** |
| Results | | |
| Participants | X | (a) Report numbers of individuals at each stage of study—eg numbers potentially eligible, examined for eligibility, confirmed eligible, included in the study, completing follow-up, and analysed  **Results: *Patient characteristics* and Figure 2** |
|  |  | (b) Give reasons for non-participation at each stage  **Results: *Patient characteristics* and Figure 2** |
|  |  | (c) Consider use of a flow diagram  **Figure 2** |
| Descriptive data | X | (a) Give characteristics of study participants (eg demographic, clinical, social) and information on exposures and potential confounders  **Results: *Patient characteristics* and Table 1. See also: Supporting information S3 Table** |
|  |  | (b) Indicate number of participants with missing data for each variable of interest  **Table 1** |
|  |  | (c) Summarise follow-up time (eg, average and total amount)  **Methods: *Selection of GTZ users and the comparison group* Results: *Patient characteristics* and Table 1** |
| Outcome data | X | Report numbers of outcome events or summary measures over time  **Results: *Results primary analysis* and Table 2** |
| Main results | X | (*a*) Give unadjusted estimates and, if applicable, confounder-adjusted estimates and their precision (eg, 95% confidence interval). Make clear which confounders were adjusted for and why they were included  **Results: *Results primary analysis* and Table 3** |
|  |  | (*b*) Report category boundaries when continuous variables were categorized  **N/a for main results. For potential confounders see Table 1** |
|  |  | (*c*) If relevant, consider translating estimates of relative risk into absolute risk for a meaningful time period  **Results: *Results primary analysis* and Table 2** |
| Other analyses | X | Report other analyses done—eg analyses of subgroups and interactions, and sensitivity analyses  **Results: *Results primary analysis , Results secondary analyses and Results sensitivity analyses*** |
| Discussion | | |
| Key results | X | Summarise key results with reference to study objectives  **Discussion: Paragraph 1** |
| Limitations | X | Discuss limitations of the study, taking into account sources of potential bias or imprecision. Discuss both direction and magnitude of any potential bias  **Discussion: *Strengths and limitations*** |
| Interpretation | X | Give a cautious overall interpretation of results considering objectives, limitations, multiplicity of analyses, results from similar studies, and other relevant evidence  **Discussion: *Conclusion*** |
| Generalisability | X | Discuss the generalisability (external validity) of the study results  **Discussion: *Strengths and limitations*** |
| Other information | | |
| Funding | x | Give the source of funding and the role of the funders for the present study and, if applicable, for the original study on which the present article is based |

*Give information separately for exposed and unexposed groups.

**Note:** An Explanation and Elaboration article discusses each checklist item and gives methodological background and published examples of transparent reporting. The STROBE checklist is best used in conjunction with this article (freely available on the Web sites of PLoS Medicine at http://www.plosmedicine.org/, Annals of Internal Medicine at http://www.annals.org/, and Epidemiology at http://www.epidem.com/). Information on the STROBE Initiative is available at http://www.strobe-statement.org.
