# Supplementary material for: Glitazone Treatment and Incidence of Parkinson’s Disease among People with Diabetes: A Retrospective Cohort Study
Source: PLoS Med. 2015 Jul 21;12(7):e1001854. doi: 10.1371/journal.pmed.1001854 (PMC4511413; doi:10.1371/journal.pmed.1001854)
Supplement: S1 Protocol — Protocol for research using the CPRD. (DOC) [file pmed.1001854.s002.doc]

# ISAC APPLICATION FORM

# PROTOCOLS FOR RESEARCH USING THE CLINICAL PRACTICE RESEARCH DATALINK (CPRD)

| ISAC use only:  Protocol Number  Date submitted | .............................  ............................. | **IMPORTANT**  **If you have any queries, please contact ISAC Secretariat:** ISAC[@cprd.com](mailto:Annalisa.Rubino@gprd.com) |
| --- | --- | --- |

| 1. Study Title   Glitazone Antidiabetics and the Risk of Parkinson’s Disease |
| --- |
| 1. Principal Investigator (full name, job title, organisation & e-mail address for correspondence regarding this protocol)   Ian Douglas, Lecturer in Pharmacoepidemiology, London School of Hygiene & Tropical Medicine, [ian.douglas@lshtm.ac.uk](mailto:ian.douglas@lshtm.ac.uk) |
| 1. Affiliation (full address)   LSHTM, Keppel St, London, WC1E 7HT |
| 1. Protocol’s Author (if different from the principal investigator) |
| 1. Type of Institution (please tick one box below)   Academia **x** Research Service Provider  Pharmaceutical Industry  NHS  Government Departments  Others |
| 1. Financial Sponsor of study   Pharmaceutical Industry *(please specify)*        Academia*(please specify)* X LSHTM  Government / NHS *(please specify)*        None  Other *(please specify)* |
| 1. Data source *(please tick one box below)*     Sponsor has on-line access **X** Purchase of ad hoc dataset  Commissioned study  Other  *(please specify)* |
| 1. Has this protocol been peer reviewed by another Committee?   Yes* No X  ** Please state in your protocol the name of the reviewing Committee(s) and provide an outline of the review process and outcome.* |
| 1. Type of Study *(please tick all the relevant boxes which apply)*   Adverse Drug Reaction/Drug Safety Drug Use  Disease Epidemiology  Drug Effectiveness X Pharmacoeconomic  Other |
| 1. This study is intended for:   Publication in peer reviewed journals X Presentation at scientific conference **x**  Presentation at company/institutional meetings **x** Other |

| 1. **Does this protocol also seek access to data held under the CPRD Data Linkage Scheme?**   **Yes**  **No X** |
| --- |
| 1. **If you are seeking access to data held under the CPRD Data Linkage Scheme, please select the source(s) of linked data being requested.**   **Hospital Episode Statistics**  **Cancer Registry Data***  **MINAP**  **ONS Mortality Data**  **Index of Multiple Deprivation/ Townsend Score**  **Mother Baby Link**  **Other: (please specify)**  ****Please note that applicants seeking access to cancer registry data must provide consent for publication of their study title and study institution on the UK Cancer Registry website. Please contact the CPRD Research Team on +44 (20) 3080 6383 or email*** [***kc@cprd.com***](mailto:kc@cprd.com) ***to discuss this requirement further.*** |
| 1. **If you are seeking access to data held under the CPRD Data Linkage Scheme, have you already discussed your request with a member of the Research team?**   **Yes**  **No***  ****Please contact the CPRD Research Team on +44 (20) 3080 6383 or email*** [***kc@cprd.com***](mailto:kc@cprd.com) ***to discuss your requirements before submitting your application.***  **Please list below the name of the person/s at the CPRD with whom you have discussed your request.** |
| 1. **Does this protocol involve requesting any additional information from GPs?**   **Yes***  **No x**  *** Please indicate what will be required:**  **Completion of questionnaires by the GP*^ψ^* Yes**  **No**  **Provision of anonymised records (e.g. hospital discharge summaries) Yes**  **No**  **Other (please describe)**  ***^ψ^ Any questionnaire for completion by GPs or other health care professional must be approved by ISAC before circulation for completion.*** |
| 1. **Does this protocol describe a purely observational study using CPRD data (this may include the review of anonymised free text)?**   **Yes* X No****  ***Yes: If you will be using data obtained from the CPRD Group, this study does not require separate ethics approval from an NHS Research Ethics Committee.***  **** No: You may need to seek separate ethics approval from an NHS Research Ethics Committee for this study. The ISAC will provide advice on whether this may be needed.*** |
| 1. **Does this study involve linking to patient *identifiable* data from other sources?**   **Yes**  **No X** |
| 1. **Does this study require contact with patients in order for them to complete a questionnaire?**   **Yes**  **No X**  ***N.B. Any questionnaire for completion by patients must be approved by ISAC before circulation for completion.*** |
| 1. **Does this study require contact with patients in order to collect a sample?**   **Yes***  **No X**  **** Please state what will be collected*** |
| 1. Experience/expertise available   Please complete the following questions to indicate the experience/expertise available within the team of researchers actively involved in the proposed research, including analysis of data and interpretation of results |
| Previous GPRD/CPRD Studies Publications using GPRD/CPRD data  None  1-3  > 3 **X** **X** |
| Yes No  Is statistical expertise available within the research team? X  *If yes, please outline level of experience* *Advanced*  Is experience of handling large data sets (>1 million records)  available within the research team? x  *If yes, please outline level of experience* Advanced  Is UK primary care experience available within the research team? X  *If yes, please outline level of experience* *Liam Smeeth is a practicing UK GP* |
| 1. References relating to your study   Please list up to 3 references (most relevant) relating to your proposed study.        Combs CK, Johnson DE, Karlo JC, Cannady SB, Landreth GE. (2000) Inflammatory mechanisms in Alzheimer's disease: inhibition of beta-amyloid-stimulated proinflammatory responses and neurotoxicity by PPARgamma agonists. J Neurosci 20(2):558-67.  Sadeghian M; Marinova-Mutafchieva L; Broom L; Davis JB; Virley D; Medhurst AD; Dexter DT. (15 May 2012). Full and partial peroxisome proliferation-activated receptor-γ agonists, but not δ agonist, rescue of dopaminergic neurons in the 6-OHDA parkinsonian model is associated with inhibition of microglial activation and MMP expression. J Neuroimmunol. 246:69-77. |
| 1. List of all investigators/collaborators (*please list the names, affiliations and e-mail addresses* of all collaborators*, other than the principal investigator)     Investigators: Dr Ian Douglas^1^, Dr Krishnan Bhaskaran^1^, Prof Nishi Chaturvedi^2^, Prof David Dexter^3^, Prof Liam Smeeth^1^:  1. Epidemiology and Population Health, London School of Hygiene & Tropical Medicine  2. National Heart and Lung Institute, Imperial College London  3. Department of Medicine, Imperial College London  ian.douglas@lshtm.ac.uk, krishnan.bhaskaran@lshtm.ac.uk, n.chaturvedi@imperial.ac.uk d.dexter@imperial.ac.uk, liam.smeeth@lshtm.ac.uk      **Please note that your ISAC application form and protocol* ***must*** *be copied to all e-mail addresses listed above at the time of submission of your application to the ISAC mailbox. Failure to do so will result in delays in the processing of your application.* |

**Protocol content checklist**

In order to help ensure that protocols submitted for review contain adequate information for protocol evaluation, ISAC have produced instructions on the content of protocols for research using CPRD data. These instructions are available on the CPRD website ([www.cprd.com/ISAC](http://www.cprd.com/ISAC)). All protocols using CPRD data which are submitted for review by ISAC must contain information on the areas detailed in the instructions. IF you do not feel that a specific area required by ISAC is relevant for your protocol, you will need to justify this decision to ISAC.

Applicants must complete the checklist below to confirm that the protocol being submitted includes all the areas required by ISAC, or to provide justification where a required area is not considered to be relevant for a specific protocol. Protocols will not be circulated to ISAC for review until the checklist has been completed by the applicant.

**Please note, your protocol will be returned to you if you do not complete this checklist, or if you answer ‘no’ and fail to include justification for the omission of any required area.**

|  | **Included in protocol?** | |  |
| --- | --- | --- | --- |
| **Required area** | **Yes** | **No** | **If no, reason for omission** |
| ***Lay Summary (max.200 words)*** | **x** |  |  |
| ***Background*** | **x** |  |  |
| ***Objective, specific aims and rationale*** | **x** |  |  |
| ***Study Type***  ***Descriptive***  ***Hypothesis Generating***  ***Hypothesis Testing*** | **x** |  |  |
| ***Study Design*** | **x** |  |  |
| ***Sample size/power calculation***  ***(Please provide justification of***  ***sample size in the protocol)*** | **x** |  |  |
| ***Study population***  ***(including estimate of expected number of***  ***relevant patients in the CPRD)*** | **x** |  |  |
| ***Selection of comparison group(s) or controls*** | **x** |  |  |
| ***Exposures, outcomes and covariates***  ***Exposures are clearly described***  ***Outcomes are clearly described*** | **x**  **x**  **x** |  |  |
| ***Data/ Statistical Analysis Plan***  ***There is plan for addressing confounding***  ***There is a plan for addressing missing data*** | **x**  **x**  **x** |  |  |
| ***Patient/ user group involvement ^†^*** | **x** |  |  |
| ***Limitations of the study design, data sources***  ***and analytic methods*** | **x** |  |  |
| ***Plans for disseminating and communicating study results*** | **x** |  |  |

***^†^ It is expected that many studies will benefit from the involvement of patient or user groups in their planning and refinement, and/or in the interpretation of the results and plans for further work. This is particularly, but not exclusively true of studies with interests in the impact on quality of life. Please indicate whether or not you intend to engage patients in any of the ways mentioned above.***

*ISAC strongly recommends that researchers using CPRD consider registering as a NRR data provider in order that others engaged in research within the UK can be made aware of current works. The* ***National Research Register (NRR)*** *is a register of ongoing and recently completed research projects funded by, or of interest to, the United Kingdom's National Health Service. Information on the NRR is available on* [*www.nrr.nhs.uk*](http://www.nrr.nhs.uk/) *.*

***Please Note: Registration with the NRR is entirely voluntary and will not replace information on ISAC approved protocols that are published in summary minutes or in the ISAC annual report****.*

**TITLE: Glitazone Antidiabetics and the Risk of Parkinson’s Disease**

**Investigators: Dr Ian Douglas^1^, Dr Krishnan Bhaskaran^1^, Prof Nishi Chaturvedi^2^, Prof David Dexter^3^, Prof Liam Smeeth^1^**:

1. Epidemiology and Population Health, London School of Hygiene & Tropical Medicine
2. National Heart and Lung Institute, Imperial College London
3. Department of Medicine, Imperial College London

[ian.douglas@lshtm.ac.uk](mailto:ian.douglas@lshtm.ac.uk), [krishnan.bhaskaran@lshtm.ac.uk](mailto:krishnan.bhaskaran@lshtm.ac.uk), [n.chaturvedi@imperial.ac.uk](mailto:n.chaturvedi@imperial.ac.uk) [d.dexter@imperial.ac.uk](mailto:d.dexter@imperial.ac.uk), [liam.smeeth@lshtm.ac.uk](mailto:liam.smeeth@lshtm.ac.uk)

**Summary of Research in plain English**

Parkinson’s disease (PD) is a progressive neurological disorder in which the brain is affected by a number of disease processes, one of which is inflammation. Recent *in vitro* and animal experiments suggest that a class of medication currently used to treat diabetes, glitazones (GTZs), may also be effective against the onset and progression of PD by reducing the inflammation in the brain associated with the disease process. GTZs have been used for >10 years in the United Kingdom to treat diabetes, and we believe that they may have slowed or prevented the onset of PD in people who have taken them. We will therefore look at a cohort of people with diabetes and compare people taking GTZs with people taking other diabetes treatments to see if those receiving a GTZ were less likely to develop PD. The findings of this study will help determine whether GTZs and drugs with similar actions may be useful in the treatment of PD.

**Aim**

To assess the effect of glitazones on the risk of Parkinson's disease.

**Objectives**

To estimate the rate ratio for Parkinson’s disease in people with diabetes, comparing individuals treated with glitazones to people treated with agents other than glitazones.

**Background & Rationale**

Parkinson’s disease is a degenerative neurological disease primarily characterised by nerve cell death in the substantia nigra and a consequent deficit in the amount of dopamine available in the central nervous system. People with PD gradually develop movement-related symptoms, including tremor, bradykinesia and rigidity. The pathophysiological causes of PD are complex, and are thought to involve oxidative stress, mitochondrial and proteasomal dysfunction and neuroinflammation (Jenner & Olanow, 2006). Neuroinflammation is partly mediated through microglia, which have been shown to proliferate and become chronically activated in PD. Microglia produce pro-inflammatory cytokines which are associated with tissue damage and are likely to partly explain the loss of dopaminergic neurons in PD (McGeer et al 1988, Forno 1992, Harada et al 1995, Liu & Hong 2003, Wyss-Coray & Mucke L 2002, Merrill & Benveniste 1996).

*In vitro* studies have shown that peroxisome proliferation-activated receptor gamma (PPARɣ agonist medications such as the antidiabetes GTZ drugs (pioglitazone and rosiglitazone) may have a neuroprotective effect through multiple anti-inflammatory properties, including an inhibitory effect on the activation of microglia (Hetzel et al 2003, Zafiriou et al 2005, Combs et al 2000). Pioglitazone specifically, has been shown to reduce inflammatory markers and offer near complete protection against dopaminergic neuronal loss in an animal model of PD (Sadeghian et al, 2012).

Current treatments for PD have focused on supplementing or improving the availability of dopamine, but such strategies are associated with diminishing returns and unpleasant side effects. So far, no effective treatments have been found to directly tackle the neurodegenerative aspect of the disease and further clinical exploration of these exciting findings with PPARγ agonists is warranted. A very small clinical trial is currently exploring the use of pioglitazone to treat PD (Clinicaltrials.gov reg: NCT01280123), but will have very limited power. Since the late 1990’s GTZs have been prescribed in patients with diabetes. The Clinical Practice Research Datalink (CPRD) offers an ideal source of observational data to examine whether GTZ antidiabetic medications are protective against PD by determining their effect on PD onset in a large population. The results of this study will add substantially to the evidence base regarding the effects of GTZs on PD in clinical practice, and will be of great value when making decisions about where to focus resources for further clinical research.

**Study Type**

This is a hypothesis testing study

**Study design**

We will conduct a cohort study comparing the rate of PD diagnosis in people treated with GTZ antidiabetic drugs, compared with people being treated with non-GTZ antidiabetic drugs. This design will minimise confounding because both exposed and unexposed patients will have diabetes and will therefore have similar underlying health problems related to diabetes which may be associated with PD. Due to the cohort design we will also be able to examine whether any effect of GTZs changes over time.

**Study Population**

The study population will be drawn from the entire CPRD population with acceptable up to standard (UTS) follow up time. People will be selected for inclusion if they have received any prescription for a GTZ antidiabetic (code list in Appendix 1), with the first occurrence being at least 12 months after the patient’s start of UTS follow up. This is to increase the likelihood that we are studying new users of GTZs, which will be of importance when we assess any effect of treatment duration on PD risk. Eligibility will begin in 1999 when the first GTZ was launched. No age restrictions will be applied to eligibility; the mean age of type 2 diabetes diagnosis in a US population was ~ 46 years in 2000 (Koopman et al, 2005) and so we anticipate the age profile of patients receiving GTZ antidiabetics will be a little younger than the typical onset age for PD (over 50 year), making them an ideal group in which to study the long term effects of a potentially neuroprotective agent. Potential participants with any diagnosis of PD prior to their first GTZ prescription will be excluded. Users will be classified according to their diabetes treatment stage at the time the GTZ is started, as follows:

1. GTZ monotherapy, no prior oral anti-diabetic (OAD)
2. GTZ monotherapy, previous OAD discontinued
3. Combined OAD therapy, GTZ being taken with other OADs

A feasibility count shows there are ~43,000 incident GTZ users with at least 12 months prior UTS follow up. Follow up for the study analysis will begin at the first prescription for a GTZ and end at the earliest of last collection date for the practice, patient transfer out or death date.

**Selection of Comparison Group**

Each GTZ exposed patient will be matched by age, sex, and practice with up to 5 patients receiving other oral treatment for diabetes (unexposed group). To reduce the possibility of confounding, we will only select patients who have also received oral antidiabetic treatments, thus reducing the difference in disease stage between GTZ users and non-users. GTZ prescribing has evolved during >10 years according to changes in treatment guidance and changes made to the licensed indication. Initially they were mostly used as second line combination treatment, but were subsequently also used at earlier stages of diabetes treatment. Rosiglitazone was eventually suspended due to cardiovascular safety concerns and pioglitazone use has waned due to concerns over the risk of bladder cancer. To be considered for inclusion, only patients present in the database from 1999 onwards and with at least 12 months UTS follow up prior to prescribing events that trigger eligibility will be included. For instance a patient could be matched to a GTZ user in category 1) above if they had never received an OAD before 1999 and began taking a sulphonylurea with no other OAD after at least 12 months UTS follow up. Follow up for the unexposed will begin at the first prescribing event that makes them eligible for inclusion.

**Power Calculation**

Amongst the 43,000 GTZ exposed patients identified for the feasibility count, 144 were first diagnosed with PD after starting a GTZ. Using STATA 12, we estimate we will have 99% power to detect a 30% reduction in PD incidence amongst GTZ users, if we match each exposed patient to five unexposed. Power to detect a 25% and 20% reduction will be and 91% and 71% respectively. We believe an effect size of 25% would represent a clinically meaningful association, and may also be plausible, given the near complete protection identified in animal studies (Sadeghian et al, 2012).

**Exposures, Outcomes and Covariates**

Exposure will be determined by prescribing records, using code lists for individual OADs. Code lists for GTZs can be found in Appendix 1. The index date for each patient will be their first prescribing event that qualifies them for study entry (e.g. first exposure to a GTZ)

The outcome for this study is the first recording of a PD diagnosis after the index date, as identified from clinical and referral records, using the Read code list in Appendix 2. PD due to known causes will be excluded based on the recording of codes presented in Appendix 2.

Covariates to be explored due to their possible association with PD and potential confounding nature will be:

- Smoking status determined using the additional clinical details file
- HRT use determined using prescribing records
- Head trauma as determined by Read code recording
- Age and sex will be accounted for through matching
- Calcium channel blocker use as determined by prescribing records: This exposure has been shown to reduce the incidence of PD (Becker et al, 2008).
- Diabetes itself has been shown to be possibly associated with PD (Wahlqvist et al, 2012). Length of time between diabetes diagnosis and index date, and HbA1c level at index date will be determined and explored for possible confounding properties. In addition, the effect of time updated HbA1c levels will be explored.
- Metformin has been suggested to also have a protective effect against PD (Wahlqvist et al, 2012). As a very commonly prescribed diabetes medication, this will be explored in detail as described below under “Statistical Analysis”.

Other covariates that have not been shown to be associated with PD will also be explored in case of possible confounding, and will include:

- Alcohol consumption
- Body mass index
- Calendar year

**Statistical Analysis**

We will measure an incidence rate ratio for the association between GTZ use and incident PD using poisson regression, comparing patients exposed to GTZs with patients exposed to alternative treatments. The primary analysis will follow all patients from their index date until the earliest of a PD record, transfer out/death, or last collection date for the practice, and their exposure will remain as defined at the index date, regardless of any subsequent changes in therapy (analogous to an intention to treat analysis in a clinical trial). Secondary analyses will be as follows:

- Censoring follow up at end of GTZ therapy, defined as last prescription plus 90 days, followed by at least 180 days GTZ-free follow up time.
- Stratified analysis based on metformin and GTZ treatment status, to explore the individual and any additive effects of both treatments. Whilst we anticipate most patients will be exposed to metformin, we anticipate a sizeable population who will not receive metformin.
- We have chosen to measure the incidence of PD from the start of therapy with GTZs as the pre-clinical work to date suggests a fast onset of any protective effect. Whether this effect varies with exposure length will be assessed through a stratified analysis on length of follow up after starting GTZ therapy.
- Sensitivity analysis defining PD as a Readcode indicating PD plus at least 2 prescriptions for an anti-Parkinson’s drug, to reduce the likelihood of outcome misclassification. Although cases with little follow up beyond their diagnosis will be excluded, this approach will only reduce study power, but will reduce misclassification bias. The date of earliest recorded diagnosis will remain the date of the outcome.
- Sensitivity analysis to look separately at the effects of pioglitazone and rosiglitazone.
- Sensitivity analysis adjusting for all variables identified as potential confounders, in addition to those included in the forward stepwise modelling strategy (see below).

Adjustments for potential confounding will be made in a forward stepwise manner retaining variables in the model if they have a substantial impact on the estimated rate ratio (a change of 10% or more), and starting with variables that have the greatest effect on PD incidence in univariate analyses. All data management and analyses will be performed using Stata 12 (StataCorp, Texas).

**Patient or User Group Involvement**

We do not believe this research would benefit from patient or user group involvement at the current stage, but may be more relevant at the time of dissemination or consideration of further related research.

**Limitations of the Study Design, Data Sources and Analytic Methods**

Diagnoses of PD in the CPRD have a positive predictive value of 90% (Becker et al, 2008) and so a small degree of misclassification amongst outcomes is expected. However, this is likely to be non-differential with respect to GTZ exposure and would be expected to bias results towards the null. In addition, missing cases of PD are likely to be rare in our study population, given that the prevalence of PD is low in the general population. Misclassification of the exposure is also likely to occur to some degree, with patients not being completely adherent to prescribed medicines. However, diabetes is a serious condition for which patients are likely to be motivated to take their medication, and substantial misclassification is not anticipated. Again, misclassification is unlikely to be differential with respect to PD status and so a dilution of any effect towards the null would be expected.

Whilst a small number of risk factors for PD are known and will be accounted for in the design and analysis (e.g. age, sex, smoking), it is not anticipated that these factors will be strongly associated with the choice to prescribe a GTZ, and so confounding is not expected to be strong in this study. Nonetheless, the analysis plan ensures potential confounding will be addressed, and in particular the separate and potentially additive effects of GTZs and metformin will be assessed.

Since the study is conducted entirely within the population with diabetes, the generalisability of results may be affected. However, it is unlikely that any pharmacological effect of GTZs relevant to PD would be limited to people with diabetes only.

**Plans for Dissemination and Communication of Study Results**

The results will be published in a relevant medical journal and will be presented at scientific meetings where possible

**References**

Becker C, Jick SS, Meier CR. Use of antihypertensives and the risk of Parkinson disease. Neurology 2008; 70: 1438-44.

Combs CK, Johnson DE, Karlo JC, Cannady SB, Landreth GE. (2000) Inflammatory mechanisms in Alzheimer's disease: inhibition of beta-amyloid-stimulated proinflammatory responses and neurotoxicity by PPARgamma agonists. J Neurosci 20(2):558-67.

Forno LS (1992). Neuropathologic features of Parkinson's, Huntington's, and Alzheimer's diseases. *Ann N Y Acad Sci*. 648:6-16.

Harada M, et al (1995).Transforming growth factor-beta 1 levels are elevated in the striatum and in ventricular cerebrospinal fluid in Parkinson's disease. *Neurosci Lett*. 193(2):129-32.

Hetzel M, Walcher D, Grüb M, Bach H, Hombach V, Marx N. (2003) Inhibition of MMP-9 expression by PPARgamma activators in human bronchial epithelial cells. Thorax. 58(9):778-83.

Jenner P, Olanow CW (2006). The pathogenesis of cell death in Parkinson's disease. Neurology. 66(10 Suppl 4):S24-36.

Koopman RJ, Mainous AG 3rd, Diaz VA, Geesey ME. Changes in age at diagnosis of type 2 diabetes mellitus in the United States, 1988 to 2000. Ann Fam Med. 2005 Jan-Feb;3(1):60-3.Liu B, Hong JS (2003). Role of microglia in inflammation-mediated neurodegenerative diseases: mechanisms and strategies for therapeutic intervention *J Pharmacol Exp Ther*. 304(1):1-7.

McGeer PL, Itagaki S, Boyes BE, McGeer EG (1988). Reactive microglia are positive for HLA-DR in the substantia nigra of Parkinson's and Alzheimer's disease brains. *Neurology*. 38(8):1285-91.

Merrill JE, Benveniste EN (1996). Cytokines in inflammatory brain lesions: helpful and harmful *Trends Neurosci*. 19(8):331-8.

Sadeghian M; Marinova-Mutafchieva L; Broom L; Davis JB; Virley D; Medhurst AD; Dexter DT. (15 May 2012). Full and partial peroxisome proliferation-activated receptor-γ agonists, but not δ agonist, rescue of dopaminergic neurons in the 6-OHDA parkinsonian model is associated with inhibition of microglial activation and MMP expression. J Neuroimmunol. 246:69-77.

Wahlqvist ML, Lee MS, Hsu CC, Chuang SY, Lee JT, Tsai HN. Metformin-inclusive sulfonylurea therapy reduces the risk of Parkinson's disease occurring with Type 2 diabetes in a Taiwanese population cohort. Parkinsonism Relat Disord. 2012 Jul;18(6):753-8. doi: 10.1016/j.parkreldis.2012.03.010. Epub 2012 Apr 10.

Wyss-Coray T, Mucke L (2002). Inflammation in neurodegenerative disease--a double-edged sword. *Neuron*. 35(3):419-32.

Zafiriou S, Stanners SR, Saad S, Polhill TS, Poronnik P, Pollock CA. (2005) Pioglitazone inhibits cell growth and reduces matrix production in human kidney fibroblasts. J Am Soc Nephrol16(3):638-45.

**Appendix 1: Codes used to identify glitazone exposure**

prodcode drugsubstancename

469 rosiglitazone maleate

548 pioglitazone hydrochloride

5227 rosiglitazone maleate

6855 metformin hydrochloride/rosiglitazone maleate

7325 metformin hydrochloride/rosiglitazone maleate

7375 metformin hydrochloride/rosiglitazone maleate

9662 rosiglitazone maleate

9699 pioglitazone hydrochloride

10051 pioglitazone hydrochloride

11601 metformin hydrochloride/rosiglitazone maleate

11604 metformin hydrochloride/rosiglitazone maleate

11609 metformin hydrochloride/rosiglitazone maleate

11610 metformin hydrochloride/rosiglitazone maleate

11717 metformin hydrochloride/rosiglitazone maleate

11737 metformin hydrochloride/rosiglitazone maleate

11760 metformin hydrochloride/rosiglitazone maleate

13628 troglitazone

14164 metformin hydrochloride/rosiglitazone maleate

15232 rosiglitazone maleate

17580 metformin hydrochloride/rosiglitazone maleate

18220 metformin/pioglitazone

19472 pioglitazone hydrochloride

20287 pioglitazone hydrochloride

20889 pioglitazone hydrochloride

30316 metformin/pioglitazone

31077 metformin/pioglitazone

37617 rosiglitazone maleate

**Appendix 2: Codes used to identify Parkinson’s Disease**

Parkinson’s disease with no identified cause

| medcode | readterm |
| --- | --- |
| 1691 | paralysis agitans |
| 4321 | parkinson's disease |
| 8956 | parkinsonism with orthostatic hypotension |
| 9509 | [x]dementia in parkinson's disease |
| 10718 | o/e - parkinsonian tremor |
| 14912 | parkinson's disease nos |
| 16860 | o/e - parkinson gait |
| 17004 | o/e - parkinson posture |
| 53655 | o/e -parkinson flexion posture |
| 59824 | o/e-festination-parkinson gait |
| 86062 | [x]parkinsonism in diseases classified elsewhere |
| 96860 | cerebral degeneration in parkinson's disease |

Parkinsonism with identified causes

| medcode | readterm |
| --- | --- |
| 19478 | drug induced parkinsonism |
| 24001 | secondary parkinsonism due to other external agents |
| 26181 | secondary parkinsonism, unspecified |
| 33544 | parkinsonism secondary to drugs |
| 51105 | postencephalitic parkinsonism |
| 52589 | syphilitic parkinsonism |
| 72879 | [x]secondary parkinsonism, unspecified |
| 97170 | [x]other secondary parkinsonism |
| 100128 | vascular parkinsonism |
