# Supplementary material for: Glitazone Treatment and Incidence of Parkinson’s Disease among People with Diabetes: A Retrospective Cohort Study
Source: PLoS Med. 2015 Jul 21;12(7):e1001854. doi: 10.1371/journal.pmed.1001854 (PMC4511413; doi:10.1371/journal.pmed.1001854)
Supplement: S1 Table — (DOC) [file pmed.1001854.s003.doc]

**S1. Table - Patient Demographics and Characteristics - Individuals diagnosed with PD *vs* Individuals not diagnosed with PD**

| **Demographics, characteristics** | **Incident diagnosis of Parkinson’s disease (N=692)** | **No Parkinson’s disease (N=164,278)** |
| --- | --- | --- |
| **Median age (quartiles)**  **Range (years)** | 70. 3 (63.9-75.1)  24.1-92.2 | 63.4 (54.2-71.7)  7.3 – 100.3 |
| **Sex, n (%)**  Female  Male | 261 (37.7)  431 (62.3) | 71,459 (43.5)  92,819 (56.5) |
| **Calendar year at indexdate**  1999-2003  2004-2008  2009-2013 | 384 (55.5)  240 (34.7)  68 (9.8) | 53,619 (32.6)  63,321 (38.5)  47,338 (28.8) |
| **Alcohol use at entry study, n (%)**  No  Yes  Ex  Excessive  Unknown | 132 (19.1)  456 (65.9)  39 (5.6)  34 (4.9)  31 (4.5) | 27,657 (16.8)  107,458 (65.4)  10,903 (6.6)  9,479 (5.8)  8,781 (5.4) |
| **Smoking at entry study, n (%)**  Current  Ex smoker  Never  Unknown | 87 (12.6)  360 (52.0)  236 (34.1)  9 (1.3) | 29,727 (18.1)  78,164 (47.6)  53,932 (32.8)  2,455 (1.5) |
| **Body mass index (kg/m^2^) at entry study, n (%)**  < 18.5  18.5 – 24.9  25.0 – 29.9  >= 30  Unknown | 1 (0.1)  133 (19.2)  246 (35.6)  293 (42.3)  19 (2.8) | 706 (0.4)  23,641 (14.4)  55,265 (33.6)  79,622 (48.5)  5,044 (3.1) |
| **Type of user, n (%)**  First line monotherapy  Second line monotherapy  Second line combination | 13 (1.9)  24 (3.5)  655 (94.6) | 5,279 (3.2)  4,926 (3.0)  154,073 (93.8) |
| **Co-morbidities, n (%)**  Head injury  Diabetes  0-2 years before entry study  2-5 years before entry study  5-9 years before entry study  >9 years before entry study  Diabetes diagnosis missing  HbA1c levels  <7.5% (57 mmol/mol)  7.6-8.3% (66 mmol/mol)  8.4-9.6% (80 mmol/mol)  >9.6%  HbA1c missing | 23 (3.3)  125 (18.1)  151 (21.8)  189 (27.3)  226 (32.7)  1 (0.1)  257 (37.1)  166 (24.0)  133 (19.2)  129 (18.6)  7 (1.0) | 5,318 (3.2)  41,015 (25.0)  41,058 (25.0)  40,936 (24.9)  40,808 (24.8)  461 (0.3)  46,074 (28.1)  38,149 (23.2)  37,316 (22.7)  40,588 (24.7)  2,151 (1.3) |
| **Co-medications, n (%)**  CCB  Never  Used >6 months before index, but not at index (past user)  Current user for less than 1 year  Current user for > 1 year  HRT  Never  Used >6 months before index, but not at index (past user)  Current user for less than 1 year  Current user for > 1 year | 413 (59.7)  153 (22.1)  24 (3.5)  102 (14.7)  647 (93.5)  29 (4.2)  1 (0.1)  15 (2.2) | 106,099 (64.6)  29,945 (18.2)  4,534 (2.8)  23,700 (14.4)  148,447 (90.4)  13,005 (7.9)  390 (0.2)  2,436 (1.5) |
