# Supplementary material for: Glitazone Treatment and Incidence of Parkinson’s Disease among People with Diabetes: A Retrospective Cohort Study
Source: PLoS Med. 2015 Jul 21;12(7):e1001854. doi: 10.1371/journal.pmed.1001854 (PMC4511413; doi:10.1371/journal.pmed.1001854)
Supplement: S1 Text — (DOC) [file pmed.1001854.s004.doc]

**S1. Codes used to identify glitazone exposure**

**Product code Name drug substance**

469 rosiglitazone maleate

548 pioglitazone hydrochloride

5227 rosiglitazone maleate

6855 metformin hydrochloride/rosiglitazone maleate

7325 metformin hydrochloride/rosiglitazone maleate

7375 metformin hydrochloride/rosiglitazone maleate

9662 rosiglitazone maleate

9699 pioglitazone hydrochloride

10051 pioglitazone hydrochloride

11601 metformin hydrochloride/rosiglitazone maleate

11604 metformin hydrochloride/rosiglitazone maleate

11609 metformin hydrochloride/rosiglitazone maleate

11610 metformin hydrochloride/rosiglitazone maleate

11717 metformin hydrochloride/rosiglitazone maleate

11737 metformin hydrochloride/rosiglitazone maleate

11760 metformin hydrochloride/rosiglitazone maleate

13628 troglitazone

14164 metformin hydrochloride/rosiglitazone maleate

15232 rosiglitazone maleate

17580 metformin hydrochloride/rosiglitazone maleate

18220 metformin/pioglitazone

19472 pioglitazone hydrochloride

20287 pioglitazone hydrochloride

20889 pioglitazone hydrochloride

30316 metformin/pioglitazone

31077 metformin/pioglitazone

37617 rosiglitazone maleate
