# Supplementary material for: Glitazone Treatment and Incidence of Parkinson’s Disease among People with Diabetes: A Retrospective Cohort Study
Source: PLoS Med. 2015 Jul 21;12(7):e1001854. doi: 10.1371/journal.pmed.1001854 (PMC4511413; doi:10.1371/journal.pmed.1001854)
Supplement: S2 Text — (DOC) [file pmed.1001854.s005.doc]

**S2. Codes used to identify Parkinson’s Disease:**

Parkinson’s disease with no identified cause

| **Medical code** | **Read term** |
| --- | --- |
| 1691 | paralysis agitans |
| 4321 | parkinson's disease |
| 8956 | parkinsonism with orthostatic hypotension |
| 9509 | [x]dementia in parkinson's disease |
| 10718 | o/e - parkinsonian tremor |
| 14912 | parkinson's disease nos |
| 16860 | o/e - parkinson gait |
| 17004 | o/e - parkinson posture |
| 53655 | o/e -parkinson flexion posture |
| 59824 | o/e-festination-parkinson gait |
| 86062 | [x]parkinsonism in diseases classified elsewhere |
| 96860 | cerebral degeneration in parkinson's disease |

Parkinson’s disease with identified causes

| **Medical code** | **Read term** |
| --- | --- |
| 19478 | drug induced parkinsonism |
| 24001 | secondary parkinsonism due to other external agents |
| 26181 | secondary parkinsonism, unspecified |
| 33544 | parkinsonism secondary to drugs |
| 51105 | postencephalitic parkinsonism |
| 52589 | syphilitic parkinsonism |
| 72879 | [x]secondary parkinsonism, unspecified |
| 97170 | [x]other secondary parkinsonism |
| 100128 | vascular parkinsonism |
