# Supplementary material for: Glitazone Treatment and Incidence of Parkinson’s Disease among People with Diabetes: A Retrospective Cohort Study
Source: PLoS Med. 2015 Jul 21;12(7):e1001854. doi: 10.1371/journal.pmed.1001854 (PMC4511413; doi:10.1371/journal.pmed.1001854)
Supplement: S3 Text — (DOCX) [file pmed.1001854.s006.docx]

**S3 Text.**

**Code list for co-morbidities and concomitant medication use as listed in Table 1.**

*Information on lifestyle, anthropometric measures and test results can be found in the CPRD *additional* and *test* files.

| Diabetes: | CCB: | HRT: | Head injury: |
| --- | --- | --- | --- |
| *medcode* | *prodcode* | *prodcode* | *medcode* |
| ----------- | ----------- | ----------- | ----------- |
| 506 | 29 | 206 | 285 |
| 608 | 71 | 256 | 403 |
| 711 | 219 | 339 | 3008 |
| 758 | 269 | 390 | 6196 |
| 1038 | 410 | 774 | 6303 |
| 1323 | 452 | 986 | 6431 |
| 1407 | 491 | 988 | 7036 |
| 1410 | 501 | 1160 | 8314 |
| 1549 | 517 | 1161 | 9740 |
| 1647 | 536 | 1183 | 10385 |
| 1682 | 541 | 1263 | 10937 |
| 1684 | 568 | 1268 | 11120 |
| 1789 | 636 | 1331 | 11681 |
| 2340 | 662 | 1488 | 12659 |
| 2342 | 700 | 1489 | 12744 |
| 2378 | 729 | 1568 | 15196 |
| 2379 | 737 | 1691 | 15860 |
| 2471 | 749 | 1760 | 16181 |
| 2472 | 793 | 1774 | 16553 |
| 2475 | 939 | 1797 | 17467 |
| 2478 | 1118 | 1804 | 18325 |
| 2664 | 1120 | 1873 | 18797 |
| 2986 | 1130 | 1922 | 24486 |
| 3286 | 1262 | 2059 | 24583 |
| 3505 | 1289 | 2111 | 24763 |
| 3550 | 1298 | 2115 | 27806 |
| 3837 | 1300 | 2140 | 28260 |
| 4513 | 1449 | 2141 | 28299 |
| 4563 | 1529 | 2208 | 32009 |
| 5002 | 1538 | 2209 | 36121 |
| 5884 | 1574 | 2262 | 40659 |
| 6125 | 1684 | 2397 | 41906 |
| 6430 | 1686 | 2402 | 42704 |
| 6509 | 1747 | 2433 | 43882 |
| 6791 | 1748 | 2452 | 44249 |
| 6813 | 1836 | 2457 | 49470 |
| 7045 | 1854 | 2728 | 52865 |
| 7059 | 1995 | 2729 | 58077 |
| 7069 | 2280 | 2766 |  |
| 7328 | 2343 | 2793 |  |
| 7563 | 2453 | 2977 |  |
| 7777 | 2521 | 3002 |  |
| 7795 | 2528 | 3036 |  |
| 8306 | 2592 | 3040 |  |
| 8403 | 2605 | 3131 |  |
| 8414 | 2663 | 3179 |  |
| 8446 | 2686 | 3387 |  |
| 8618 | 2746 | 3423 |  |
| 8836 | 2811 | 3433 |  |
| 8842 | 2888 | 3530 |  |
| 9013 | 2926 | 3553 |  |
| 9145 | 3057 | 3960 |  |
| 9310 | 3061 | 4207 |  |
| 9835 | 3118 | 4244 |  |
| 9881 | 3221 | 4326 |  |
| 9897 | 3302 | 4328 |  |
| 9974 | 3342 | 4382 |  |
| 10042 | 3343 | 4393 |  |
| 10098 | 3370 | 4466 |  |
| 10099 | 3676 | 4467 |  |
| 10278 | 3711 | 4721 |  |
| 10418 | 3712 | 4882 |  |
| 10642 | 3917 | 4909 |  |
| 10659 | 3930 | 4956 |  |
| 10692 | 3931 | 4977 |  |
| 10755 | 3943 | 5005 |  |
| 10824 | 4227 | 5012 |  |
| 10921 | 4239 | 5100 |  |
| 10977 | 4308 | 5195 |  |
| 11018 | 4408 | 5292 |  |
| 11041 | 4542 | 5755 |  |
| 11047 | 4635 | 5759 |  |
| 11050 | 4732 | 5776 |  |
| 11094 | 4808 | 5798 |  |
| 11129 | 4852 | 5931 |  |
| 11149 | 4856 | 6030 |  |
| 11348 | 4923 | 6041 |  |
| 11359 | 4939 | 6059 |  |
| 11433 | 5054 | 6082 |  |
| 11471 | 5158 | 6177 |  |
| 11551 | 5162 | 6492 |  |
| 11599 | 5181 | 6538 |  |
| 11626 | 5194 | 6545 |  |
| 11663 | 5234 | 6563 |  |
| 11677 | 5277 | 6601 |  |
| 11818 | 5296 | 6603 |  |
| 11848 | 5326 | 6622 |  |
| 11930 | 5348 | 6793 |  |
| 12030 | 5477 | 6861 |  |
| 12213 | 5513 | 7001 |  |
| 12225 | 5570 | 7175 |  |
| 12247 | 5593 | 7242 |  |
| 12262 | 5806 | 7251 |  |
| 12307 | 5914 | 7381 |  |
| 12455 | 6309 | 7388 |  |
| 12483 | 6477 | 7604 |  |
| 12506 | 6510 | 7615 |  |
| 12507 | 6856 | 7619 |  |
| 12640 | 7280 | 7856 |  |
| 12675 | 7398 | 7978 |  |
| 12682 | 7541 | 8162 |  |
| 12703 | 7562 | 8465 |  |
| 12736 | 7681 | 8565 |  |
| 13057 | 7823 | 8770 |  |
| 13067 | 8024 | 8777 |  |
| 13068 | 8201 | 8837 |  |
| 13069 | 8213 | 9047 |  |
| 13070 | 8257 | 9181 |  |
| 13071 | 8310 | 9224 |  |
| 13074 | 8524 | 9235 |  |
| 13078 | 8558 | 9268 |  |
| 13097 | 8642 | 9413 |  |
| 13099 | 8759 | 9649 |  |
| 13101 | 8884 | 9671 |  |
| 13102 | 8945 | 9684 |  |
| 13103 | 8975 | 9901 |  |
| 13108 | 9094 | 9905 |  |
| 13191 | 9240 | 9976 |  |
| 13192 | 9269 | 10052 |  |
| 13194 | 9334 | 10076 |  |
| 13195 | 9374 | 10096 |  |
| 13196 | 9386 | 10105 |  |
| 13197 | 9410 | 10126 |  |
| 13279 | 9437 | 10180 |  |
| 13281 | 9485 | 10946 |  |
| 14803 | 9553 | 10967 |  |
| 14889 | 9569 | 11157 |  |
| 15690 | 9573 | 11297 |  |
| 16230 | 9670 | 11363 |  |
| 16490 | 9708 | 11375 |  |
| 16491 | 9723 | 11511 |  |
| 16502 | 9750 | 11642 |  |
| 16881 | 9919 | 11672 |  |
| 17067 | 10135 | 11859 |  |
| 17095 | 10136 | 11882 |  |
| 17236 | 10153 | 11934 |  |
| 17247 | 10246 | 12661 |  |
| 17262 | 10267 | 12713 |  |
| 17313 | 10595 | 12773 |  |
| 17478 | 10688 | 12788 |  |
| 17545 | 10832 | 12807 |  |
| 17817 | 10897 | 12907 |  |
| 17846 | 11223 | 12953 |  |
| 17858 | 11512 | 12970 |  |
| 17859 | 11547 | 13146 |  |
| 17869 | 11567 | 13582 |  |
| 17886 | 11769 | 13945 |  |
| 18056 | 11770 | 13990 |  |
| 18142 | 11777 | 14027 |  |
| 18167 | 11922 | 14569 |  |
| 18209 | 11943 | 14580 |  |
| 18219 | 11965 | 14596 |  |
| 18230 | 11966 | 14792 |  |
| 18264 | 11972 | 14812 |  |
| 18278 | 11973 | 14950 |  |
| 18311 | 12104 | 15194 |  |
| 18387 | 12392 | 15328 |  |
| 18390 | 12606 | 15869 |  |
| 18425 | 12613 | 16392 |  |
| 18496 | 12639 | 16426 |  |
| 18505 | 12705 | 16437 |  |
| 18583 | 12875 | 16493 |  |
| 18642 | 13027 | 16997 |  |
| 18662 | 13033 | 17293 |  |
| 18683 | 13075 | 17529 |  |
| 18747 | 13127 | 18009 |  |
| 18766 | 13139 | 18167 |  |
| 18777 | 13240 | 18218 |  |
| 18824 | 13243 | 18311 |  |
| 19381 | 13251 | 18383 |  |
| 19739 | 13302 | 18437 |  |
| 19781 | 13410 | 18502 |  |
| 20368 | 13672 | 18530 |  |
| 20696 | 13699 | 18600 |  |
| 20900 | 13856 | 18901 |  |
| 21420 | 13926 | 19145 |  |
| 21472 | 13965 | 19429 |  |
| 21482 | 14300 | 19432 |  |
| 21689 | 14305 | 19487 |  |
| 22023 | 14861 | 19974 |  |
| 22130 | 14892 | 19986 |  |
| 22487 | 15117 | 20155 |  |
| 22573 | 15288 | 20804 |  |
| 22823 | 15652 | 20888 |  |
| 22871 | 15659 | 21492 |  |
| 22884 | 16038 | 22741 |  |
| 22959 | 16073 | 22975 |  |
| 22967 | 16162 | 24764 |  |
| 23479 | 16328 | 24917 |  |
| 24327 | 16677 | 28230 |  |
| 24363 | 16850 | 35091 |  |
| 24405 | 17006 | 35198 |  |
| 24423 | 17325 | 35718 |  |
| 24458 | 17406 | 35742 |  |
| 24490 | 17425 | 35958 |  |
| 24571 | 17474 | 37033 |  |
| 24693 | 17492 | 37037 |  |
| 24694 | 17557 | 37692 |  |
| 24836 | 17566 | 37697 |  |
| 25041 | 17586 | 38932 |  |
| 25591 | 17599 | 38935 |  |
| 25627 | 17640 | 38940 |  |
| 25636 | 17666 | 38965 |  |
| 26054 | 18038 | 39622 |  |
| 26108 | 18223 | 40606 |  |
| 26603 | 18379 | 40643 |  |
| 26604 | 18404 | 40836 |  |
| 26605 | 18606 | 40848 |  |
| 26664 | 18830 | 44461 |  |
| 26666 | 18834 | 44494 |  |
| 26667 | 18852 | 49985 |  |
| 26855 | 18874 | 51978 |  |
| 27891 | 19129 |  |  |
| 27921 | 19170 |  |  |
| 28574 | 19175 |  |  |
| 28622 | 19426 |  |  |
| 28769 | 19440 |  |  |
| 28856 | 19690 |  |  |
| 28873 | 20257 |  |  |
| 29041 | 20311 |  |  |
| 29979 | 20459 |  |  |
| 30294 | 20579 |  |  |
| 30323 | 20591 |  |  |
| 30477 | 20642 |  |  |
| 30648 | 20878 |  |  |
| 31053 | 20890 |  |  |
| 31141 | 21145 |  |  |
| 31156 | 21162 |  |  |
| 31157 | 21216 |  |  |
| 31171 | 21245 |  |  |
| 31172 | 21763 |  |  |
| 31240 | 21795 |  |  |
| 31241 | 21872 |  |  |
| 31310 | 21886 |  |  |
| 31752 | 21918 |  |  |
| 31790 | 22019 |  |  |
| 31949 | 22142 |  |  |
| 32193 | 22217 |  |  |
| 32359 | 22241 |  |  |
| 32403 | 22619 |  |  |
| 32556 | 22696 |  |  |
| 32619 | 22826 |  |  |
| 32627 | 23233 |  |  |
| 32739 | 23458 |  |  |
| 32770 | 23733 |  |  |
| 32885 | 23736 |  |  |
| 33254 | 23805 |  |  |
| 33343 | 23823 |  |  |
| 33807 | 23872 |  |  |
| 33969 | 24228 |  |  |
| 34152 | 24365 |  |  |
| 34268 | 24366 |  |  |
| 34283 | 25026 |  |  |
| 34450 | 25132 |  |  |
| 34528 | 25572 |  |  |
| 34912 | 25777 |  |  |
| 35105 | 25919 |  |  |
| 35107 | 26265 |  |  |
| 35116 | 26267 |  |  |
| 35288 | 26269 |  |  |
| 35316 | 26270 |  |  |
| 35321 | 26309 |  |  |
| 35383 | 26337 |  |  |
| 35385 | 26460 |  |  |
| 35399 | 26674 |  |  |
| 35785 | 26774 |  |  |
| 36633 | 27136 |  |  |
| 36695 | 27401 |  |  |
| 36798 | 27685 |  |  |
| 37315 | 28438 |  |  |
| 37625 | 28688 |  |  |
| 37648 | 28721 |  |  |
| 37806 | 29145 |  |  |
| 37957 | 29676 |  |  |
| 38076 | 30197 |  |  |
| 38078 | 30199 |  |  |
| 38103 | 30242 |  |  |
| 38129 | 30473 |  |  |
| 38130 | 30557 |  |  |
| 38161 | 30915 |  |  |
| 38617 | 31337 |  |  |
| 38986 | 31761 |  |  |
| 39070 | 32089 |  |  |
| 39317 | 32262 |  |  |
| 39406 | 33932 |  |  |
| 39420 | 34093 |  |  |
| 39481 | 34101 |  |  |
| 39809 | 34115 |  |  |
| 40023 | 34146 |  |  |
| 40363 | 34475 |  |  |
| 40401 | 34581 |  |  |
| 40682 | 34824 |  |  |
| 40837 | 35096 |  |  |
| 40962 | 35173 |  |  |
| 41049 | 35174 |  |  |
| 41389 | 35189 |  |  |
| 41716 | 35304 |  |  |
| 42217 | 35317 |  |  |
| 42505 | 35329 |  |  |
| 42729 | 35343 |  |  |
| 42762 | 35592 |  |  |
| 42831 | 35646 |  |  |
| 43139 | 35697 |  |  |
| 43227 | 37025 |  |  |
| 43453 | 37184 |  |  |
| 43493 | 37774 |  |  |
| 43785 | 37897 |  |  |
| 43857 | 38545 |  |  |
| 43921 | 38632 |  |  |
| 43951 | 38634 |  |  |
| 44033 | 38818 |  |  |
| 44260 | 38831 |  |  |
| 44440 | 38855 |  |  |
| 44443 | 38865 |  |  |
| 44779 | 38876 |  |  |
| 44982 | 38882 |  |  |
| 45250 | 38964 |  |  |
| 45276 | 39171 |  |  |
| 45467 | 39298 |  |  |
| 45491 | 39357 |  |  |
| 45913 | 39800 |  |  |
| 45919 | 39984 |  |  |
| 46150 | 40316 |  |  |
| 46290 | 40405 |  |  |
| 46301 | 40639 |  |  |
| 46521 | 40668 |  |  |
| 46533 | 41203 |  |  |
| 46577 | 41205 |  |  |
| 46624 | 42731 |  |  |
| 46850 | 42819 |  |  |
| 46917 | 43410 |  |  |
| 46963 | 43430 |  |  |
| 47011 | 43753 |  |  |
| 47032 | 43818 |  |  |
| 47058 | 44192 |  |  |
| 47144 | 44859 |  |  |
| 47315 | 45051 |  |  |
| 47321 | 45685 |  |  |
| 47328 | 46355 |  |  |
| 47341 | 46687 |  |  |
| 47370 | 46715 |  |  |
| 47377 | 46724 |  |  |
| 47409 | 46884 |  |  |
| 47582 | 46887 |  |  |
| 47584 | 47002 |  |  |
| 47649 | 47230 |  |  |
| 47650 | 47415 |  |  |
| 47816 | 47467 |  |  |
| 47954 | 47530 |  |  |
| 48078 | 47573 |  |  |
| 48192 | 47608 |  |  |
| 49074 | 47616 |  |  |
| 49146 | 48272 |  |  |
| 49276 | 48282 |  |  |
| 49554 | 48288 |  |  |
| 49559 | 48457 |  |  |
| 49640 | 49001 |  |  |
| 49655 | 49289 |  |  |
| 49884 | 49338 |  |  |
| 49949 | 49390 |  |  |
| 50175 | 49762 |  |  |
| 50225 | 51489 |  |  |
| 50429 | 53220 |  |  |
| 50609 |  |  |  |
| 50813 |  |  |  |
| 50937 |  |  |  |
| 50960 |  |  |  |
| 50972 |  |  |  |
| 51261 |  |  |  |
| 51371 |  |  |  |
| 51697 |  |  |  |
| 51756 |  |  |  |
| 51957 |  |  |  |
| 52041 |  |  |  |
| 52104 |  |  |  |
| 52212 |  |  |  |
| 52236 |  |  |  |
| 52237 |  |  |  |
| 52283 |  |  |  |
| 52303 |  |  |  |
| 52409 |  |  |  |
| 52630 |  |  |  |
| 53200 |  |  |  |
| 53238 |  |  |  |
| 53392 |  |  |  |
| 53634 |  |  |  |
| 54008 |  |  |  |
| 54212 |  |  |  |
| 54600 |  |  |  |
| 54601 |  |  |  |
| 54773 |  |  |  |
| 54846 |  |  |  |
| 54856 |  |  |  |
| 54899 |  |  |  |
| 55075 |  |  |  |
| 55123 |  |  |  |
| 55140 |  |  |  |
| 55239 |  |  |  |
| 55431 |  |  |  |
| 55842 |  |  |  |
| 56448 |  |  |  |
| 56803 |  |  |  |
| 57278 |  |  |  |
| 57333 |  |  |  |
| 57389 |  |  |  |
| 57621 |  |  |  |
| 57723 |  |  |  |
| 58133 |  |  |  |
| 58159 |  |  |  |
| 58604 |  |  |  |
| 58639 |  |  |  |
| 59253 |  |  |  |
| 59288 |  |  |  |
| 59365 |  |  |  |
| 59725 |  |  |  |
| 59903 |  |  |  |
| 59991 |  |  |  |
| 60107 |  |  |  |
| 60208 |  |  |  |
| 60499 |  |  |  |
| 60796 |  |  |  |
| 61021 |  |  |  |
| 61071 |  |  |  |
| 61122 |  |  |  |
| 61210 |  |  |  |
| 61344 |  |  |  |
| 61470 |  |  |  |
| 61523 |  |  |  |
| 61670 |  |  |  |
| 61829 |  |  |  |
| 62107 |  |  |  |
| 62146 |  |  |  |
| 62209 |  |  |  |
| 62384 |  |  |  |
| 62674 |  |  |  |
| 63357 |  |  |  |
| 63371 |  |  |  |
| 63412 |  |  |  |
| 63690 |  |  |  |
| 63762 |  |  |  |
| 64142 |  |  |  |
| 64283 |  |  |  |
| 64357 |  |  |  |
| 64384 |  |  |  |
| 64446 |  |  |  |
| 64449 |  |  |  |
| 64571 |  |  |  |
| 64668 |  |  |  |
| 65025 |  |  |  |
| 65267 |  |  |  |
| 65463 |  |  |  |
| 65616 |  |  |  |
| 65684 |  |  |  |
| 65704 |  |  |  |
| 66274 |  |  |  |
| 66475 |  |  |  |
| 66872 |  |  |  |
| 66965 |  |  |  |
| 67664 |  |  |  |
| 67853 |  |  |  |
| 67905 |  |  |  |
| 68105 |  |  |  |
| 68390 |  |  |  |
| 68517 |  |  |  |
| 68546 |  |  |  |
| 68818 |  |  |  |
| 68928 |  |  |  |
| 68960 |  |  |  |
| 69043 |  |  |  |
| 69124 |  |  |  |
| 69152 |  |  |  |
| 69278 |  |  |  |
| 69676 |  |  |  |
| 69993 |  |  |  |
| 70766 |  |  |  |
| 70821 |  |  |  |
| 72320 |  |  |  |
| 72345 |  |  |  |
| 72702 |  |  |  |
| 83485 |  |  |  |
| 83532 |  |  |  |
| 85660 |  |  |  |
| 85991 |  |  |  |
| 90301 |  |  |  |
| 91164 |  |  |  |
| 91646 |  |  |  |
| 91943 |  |  |  |
| 93380 |  |  |  |
| 93390 |  |  |  |
| 93468 |  |  |  |
| 93491 |  |  |  |
| 93529 |  |  |  |
| 93530 |  |  |  |
| 93631 |  |  |  |
| 93657 |  |  |  |
| 93704 |  |  |  |
| 93727 |  |  |  |
| 93854 |  |  |  |
| 93870 |  |  |  |
| 93878 |  |  |  |
| 94011 |  |  |  |
| 94186 |  |  |  |
| 94699 |  |  |  |
| 94777 |  |  |  |
| 94955 |  |  |  |
| 94956 |  |  |  |
| 95093 |  |  |  |
| 95094 |  |  |  |
| 95159 |  |  |  |
| 95351 |  |  |  |
| 95539 |  |  |  |
| 95553 |  |  |  |
| 95636 |  |  |  |
| 95813 |  |  |  |
| 95920 |  |  |  |
| 95992 |  |  |  |
| 95994 |  |  |  |
| 96010 |  |  |  |
| 96235 |  |  |  |
| 96506 |  |  |  |
| 96942 |  |  |  |
| 97281 |  |  |  |
| 97809 |  |  |  |
| 97824 |  |  |  |
| 97849 |  |  |  |
| 97894 |  |  |  |
| 98616 |  |  |  |
| 98954 |  |  |  |
| 99628 |  |  |  |
| 100770 |  |  |  |
| 100964 |  |  |  |
| 102389 |  |  |  |
